# Comparative RNA-Seq analysis reveals pervasive tissue-specific alternative polyadenylation in *Caenorhabditis elegans* intestine and muscles

**DOI:** 10.1186/s12915-015-0116-6

**Published:** 2015-01-20

**Authors:** Stephen M Blazie, Cody Babb, Henry Wilky, Alan Rawls, Jin G Park, Marco Mangone

**Affiliations:** Molecular and Cellular Biology Graduate Program, Arizona State University, Tempe, AZ USA; Virginia G. Piper Center for Personalized Diagnostics, The Biodesign Institute at Arizona State University, 1001 S McAllister Ave, Tempe, AZ USA; Barrett Honors College, Arizona State University, 751 E Lemon Mall, 1282 Tempe, AZ USA

**Keywords:** RNA-Seq, Tissue-specific mRNA, Elegans, Promoter, Transcription, mRNA-tagging, Transcriptome, 3’UTR, miRNAs, PAT-Seq

## Abstract

**Background:**

Tissue-specific RNA plasticity broadly impacts the development, tissue identity and adaptability of all organisms, but changes in composition, expression levels and its impact on gene regulation in different somatic tissues are largely unknown. Here we developed a new method, polyA-tagging and sequencing (PAT-Seq) to isolate high-quality tissue-specific mRNA from *Caenorhabditis elegans* intestine, pharynx and body muscle tissues and study changes in their tissue-specific transcriptomes and 3’UTRomes.

**Results:**

We have identified thousands of novel genes and isoforms differentially expressed between these three tissues. The intestine transcriptome is expansive, expressing over 30% of *C. elegans* mRNAs, while muscle transcriptomes are smaller but contain characteristic unique gene signatures. Active promoter regions in all three tissues reveal both known and novel enriched tissue-specific elements, along with putative transcription factors, suggesting novel tissue-specific modes of transcription initiation. We have precisely mapped approximately 20,000 tissue-specific polyadenylation sites and discovered that about 30% of transcripts in somatic cells use alternative polyadenylation in a tissue-specific manner, with their 3’UTR isoforms significantly enriched with microRNA targets.

**Conclusions:**

For the first time, PAT-Seq allowed us to directly study tissue specific gene expression changes in an *in vivo* setting and compare these changes between three somatic tissues from the same organism at single-base resolution within the same experiment. We pinpoint precise tissue-specific transcriptome rearrangements and for the first time link tissue-specific alternative polyadenylation to miRNA regulation, suggesting novel and unexplored tissue-specific post-transcriptional regulatory networks in somatic cells.

**Electronic supplementary material:**

The online version of this article (doi:10.1186/s12915-015-0116-6) contains supplementary material, which is available to authorized users.

## Background

Transcriptome plasticity is a powerful modulator of gene expression that provides multicellular organisms with the complexity needed to drive development and maintain tissue identity. Apart from a few cases, we still do not fully understand what specific transcriptome rearrangements occur in somatic tissues, and how they integrate with gene regulation at the post-transcriptional level to maintain tissue and cellular diversity.

The small nematode *Caenorhabditis elegans* is an ideal model organism to study these events, since its gene model has been extensively characterized in past years [1]. It is also experimentally tractable with short and precise developmental timing, approximately 1,000 somatic cells, a transparent and simple body plan and an entirely defined cell lineage [2]. Large-scale efforts have detailed its transcriptome at a global level [1]. Promoter diversity [3], alternate splicing events [4] and changes in 3’ untranslated regions (3’UTRs) [5,6] are also well characterized.

While the formation of several worm tissues and the genes involved in driving these processes have been extensively described [7,8], we still do not fully understand how the synergistic activity of tissue-specific events before, during and after transcription drive and maintain tissue identity. Pre-transcriptionally, enrichment of sequence-specific elements within *C. elegans* promoters has been linked to tissue-specific changes in gene expression [9-11], suggesting that these elements, together with *trans*-acting factors that recognize them, are fundamental for driving the transcriptional programs of unique tissues.

During transcription, mRNA isoforms resulting from alternative splicing increase transcriptome complexity, coordinating tissue development [12]. Recently, a genome-wide study in *C. elegans* found that thousands of transcripts are alternatively spliced and many of them change splicing patterns during development [4], suggesting that tissue-specific splicing may play key roles in this process.

Post-transcriptionally, 3’UTRs are known to contain multiple regulatory sequence elements important for gene regulation [13]. Recently, two independent studies suggest that more than 40% of worm genes possess 3’UTRs subjected to alternative polyadenylation (APA), a mechanism that generates multiple 3’UTR isoforms for the same genes [5,6]. This process is widespread in metazoans [14,15], coordinated through development [5,6], and misregulated in disease [14], underscoring a potential role for APA in tissue-specific modulation of gene expression.

The cleavage and polyadenylation of nascent mRNAs in eukaryotes is mainly executed by two large multimeric complexes named cleavage and polyadenylation specificity factor (CPSF) and cleavage stimulation factor (CstF) [16]. CPSF recognizes and binds to the polyadenylation (poly(A)) signal (PAS) element located approximately 19 nt from the polyA site in the 3’UTR of mRNAs. In metazoans, the PAS sequence is commonly AAUAAA [16]. This sequence is necessary and sufficient for 3’end polyadenylation [16]. CstF directly interacts with CPSF and binds to GU-rich elements downstream of the cleavage site [16].

Although APA is pervasive in worms and correlated with development, suggesting that APA functions in worms tissues [5], it is unclear whether APA is tissue-specific.

Both CPSF and CstF are likely to have a role in managing the choice between PAS elements in the same 3’UTR and inducing APA. There may also be additional tissue-specific accessory factors that modify the basal polyadenylation machinery, controlling the usage of one PAS element over another. Tissue-specific isoforms of the CPSF or CstF complexes could be responsible for APA [17,18]. Over a decade ago, stoichiometric levels of CstF members were indeed shown to control APA in B cell activation [19], and recent high-throughput approaches showed that other factors might also play important roles in modulating APA [20,21]. Other processing factors were also recently shown to influence the location of cleavage [21]. These studies underscore the importance of the correct stoichiometric ratio of each of the 3’end processing factors for producing a mature mRNA. Surprisingly, it was also recently shown that U1 snRNP is involved in this process, suggesting possible cross talk between APA and the RNA splicing machinery [22]. These models may not be mutually exclusive.

In *C. elegans,* the isolation of tissue-specific mRNA to study transcriptome plasticity and APA is challenging due to the lack of *in vitro* cell cultures, the worm’s tough outer cuticle that interferes with sample preparation and the small size of many tissues that prevents manual dissection. Several techniques have been developed to circumvent these issues, including fluorescence-activated cell and nuclear sorting [23,24], nuclei-tagging [25] and mRNA-tagging [26]. In particular, mRNA-tagging has been widely used to isolate and study mRNA from muscle [27,28], epithelial [29], hypodermal [30], neuronal [31] and seam cells [28]. This technique uses tissue-specific promoters to drive expression of a FLAG epitope-tagged cytoplasmic poly-A binding protein (PABPC), which specifically binds to the poly(A)-tail of mRNAs in the cytoplasm, followed by crosslinking and immunoprecipitation of tissue-specific mRNAs. Thus far, mRNA-tagging has been coupled with DNA microarrays and genomic tiling arrays, which both lack the advanced sensitivity and specificity possible today with deep sequencing, potentially limiting the results obtained with this methodology.

Recently, a novel method that couples tissue-specific nuclei isolation with deep sequencing was used to analyze the *C. elegans* intestine transcriptome [24]. Unfortunately, while enhancing the sensitivity of tissue-specific mRNA extraction and sequencing, this technique limits the analysis to nuclear mRNA species, and only identifies short 3’end portions of 3’UTRs that need to be bioinformatically attached to their closest genes, potentially introducing mapping inaccuracies. In addition, this method does not provide tissue-specific mRNA isoform data, losing an important component needed to study the transcriptome plasticity of individual tissues.

Integrating mRNA-tagging with RNA-Seq analysis could significantly improve the resolution of these studies and identify additional factors controlling tissue development and identity. Here, we have improved tissue-specific transcriptome profiling in *C. elegans*, optimizing mRNA-tagging for deep sequencing. We call this updated method polyA-tagging and sequencing (PAT-Seq). We have applied PAT-Seq and profiled tissue-specific mRNA from the *C. elegans* intestine and two muscle tissues belonging to the pharynx and body wall of mixed stage worms. We describe and compare gene expression, promoter sequence composition, mRNA isoforms changes and alternative polyadenylation between each tissue.

PAT-Seq significantly improved the resolution of tissue-specific transcriptomes from previous studies, adding thousands of novel genes and isoforms that allow for a more comprehensive analysis of them. In addition, we have used PAT-Seq to profile the transcriptome of a previously uncharacterized tissue (pharynx), allowing us for the first time to directly compare gene expression changes between different tissues from the same organism at a higher resolution, using the same experimental settings.

We find that transcript diversity detected among these three tissues is mirrored by the presence of characteristic tissue-specific promoter signatures. In addition, we found that APA is widely used at a tissue-specific level, highlighting major complex tissue-specific transcript dynamics and post-transcriptional regulatory mechanisms. We describe a large number of 3’UTR isoforms specifically expressed in each tissue and find that these 3’UTRs are enriched for experimentally and bioinformatically predicted microRNA (miRNA) targets, suggesting that tissue-specific APA is used in worms as a mechanism to interface with miRNA mediated post-transcriptional gene regulation.

Finally, we have remapped, incorporated and curated 3’UTR data from previously published studies [1,5,6,24,32] and integrated these data with our new tissue-specific datasets. The database is accessible through our new worm APA-specific website [33], which is publically available and represents a unique resource for the scientists interested in 3’UTR biology.

## Results

### Isolation of mRNAs from intestine and muscle tissues

mRNA-tagging has so far been coupled with low-resolution platforms, such as microarrays and tiling arrays, that lack the sensitivity required to detect low expressed transcripts, pinpoint gene isoform changes and map 3’UTRs at single base resolution. To improve upon its sensitivity and specificity, we made several key changes to the original mRNA-tagging protocol [26,34]: 1) We added three tandem FLAG-epitope (3 × FLAG) tags instead of one, to improve the efficiency of the FLAG pull-down [35]. 2) In the original mRNA-tagging protocol, the FLAG-tagged PABPC construct is selectively expressed as extra chromosomal arrays, which are unstable and often lead to mosaic expression patterns, or integrated as multiple copy lines [34,35]. We instead opted for the widely used Mos-1 single copy insertion (MosSCI) technology [36], which stably incorporates the construct of interest in the worm genome. 3) We adopted a novel strategy to prepare the tissue-specific cDNA libraries that relies on linear amplification of mRNAs, minimizing the quantification error rate due to limited starting material, providing high-quality transcriptome and 3’end data in the same experiment [37]; and 4) replaced the microarray step with Next Generation sequencing (Illumina HiSeq) to improve data resolution.

**Figure 1:**
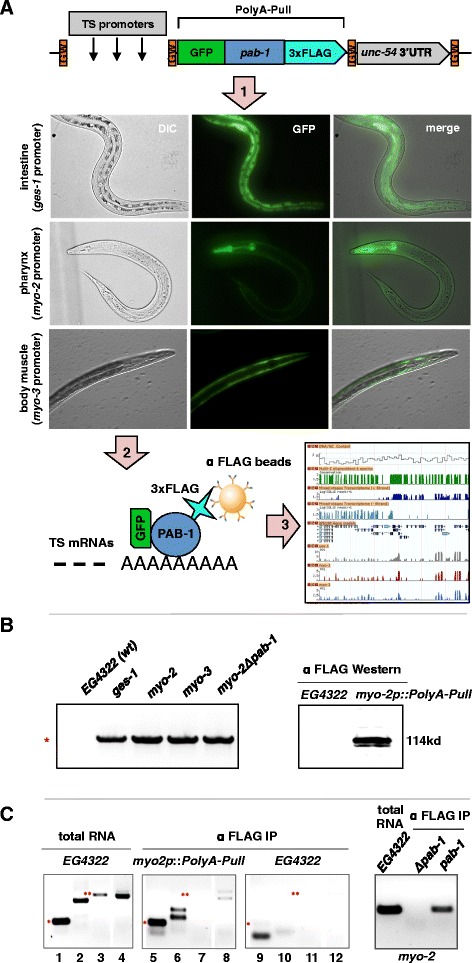
**PAT-Seq of intestine and muscles tissues. (A)** PAT-Seq uses Gateway-compatible (GW) entry vectors expressing the PolyA-Pull cassette in each tissue using tissue-specific (TS) promoters. (1) PolyA-Pull expressed in the intestine (*ges-1* promoter), pharynx (*myo-2)*, and body muscle (*myo-3*). (2) Expression of PolyA-Pull produces a 3 × FLAG-tag (light blue) fused to PAB-1 (blue), which specifically binds to the poly(A) tails of mRNAs (TS mRNAs). The complex is immunoprecipitated using α-FLAG beads. (3) Tissue-specific cDNA libraries are sequenced and mapped onto the WS190 gene model. **(B)** Detection of stable integration of the PolyA-Pull cassette. *Left panel:* Using PCR we detected genomic integration of the common portion of the PolyA-Pull cassette (2.6 kb band, red asterisk) in each tissue. The negative control, *myo-2Δpab-1,* was also integrated. *Right panel:* Western blotting using α-FLAG antibodies detected the *in-frame* PolyA-Pull fusion protein in lysates from transgenic worms expressing it in the pharynx (*myo-2::pab-1*) but not in lysate from wild type N2 worms. **(C)** Quantification of the specificity and sensitivity of the pull-down using RT-PCR: *Left panel*: *myo-2* (lane 1) (*), *ges-1* (lane 3) (**) and *dpy-7* (lane 4) transcripts were detected in total RNA extracted from wild type N2 worms. *Middle panel*: Using immunoprecipitation, we successfully detected the presence of the muscle-specific gene *myo-2* (lane 5) (*) and the exogenous *unc-54* 3’UTR (lane 6), but not the intestine-specific *ges-1* (lane 7) (**) and the hypodermis-specific *dpy-7* (lane 8). These transgenic worms expressed PolyA-Pull cassette in the pharynx, but not in our negative control in wild type N2 worms (lanes 9-12). The primers used to detect *unc-54* 3’UTR also detected 18S rRNAs (lane 2). This band was replaced with two *unc-54* 3’UTR isoforms (lane 6), suggesting that PolyA-Pull enriched for polyA+ RNAs. *Right panel:* We are unable to isolate tissue-specific RNA from worms lacking *pab-1 (Δpab-1)*.

We used tissue-specific promoters to drive the expression of the *C. elegans* cytoplasmic PABPC gene (*pab-1*), in-frame with GFP and fused to a 3 × FLAG tag (PolyA-Pull), in intestine, pharynx or body muscle (Figure 1A, see Methods). As MosSCI technology mediates transgene and rescue cassette (*unc-119*) insertion into a specified region of chromosome II, we confirmed this integration event in each transgenic worm line (Figure 1B, left panel) and verified its expression using western blotting (Figure 1B, right panel).

We tested the sensitivity and tissue-specificity of our mRNA pull-down approach using worms expressing PolyA-Pull in the pharynx (*myo-2p::PolyA-Pull*) (Figure 1C), validating the ability of our construct to selectively bind muscle specific transcripts (Figure 1C, lanes 5 and 6). The known intestine-specific transcript *ges-1* (Figure 1C, lane 7) and hypodermis-specific *dpy-7* (Figure 1C, Lane 8) were depleted from the same sample. Immunoprecipitation from wild type N2 worms yielded no detectable background in RT-PCR for the same genes (Figure 1C, lanes 9-12). To test if our PolyA-Pull construct selectively binds polyA+ RNAs, we prepared a GFP::3 × FLAG fusion protein that does not contain *pab-1*. This vector is unable to bind polyadenylated mRNAs (Δ*pab-1*-Pull, see Methods). We expressed this new construct in the pharynx (*myo-2p*::Δ*pab-1-Pull*). As expected, using this construct, we were unable to detect the pharynx-specific *myo-2* transcript (Figure 1C, *right*).

Taken together, these results suggest that our PolyA-Pull construct effectively enriches tissue-specific mRNAs and specifically binds polyA+ RNAs.

### PAT-Seq analysis of mRNAs from intestine and muscle tissues

We then prepared our tissue-specific libraries with two biological replicates (six preparations in total). We also performed two negative control pull-down experiments expressing the Δ*pab-1*-Pull construct in the pharynx to optimize the sensitivity and specificity of our approach. Following the pull-down, the cDNA libraries were prepared using isothermal linear cDNA amplifications, which allows cDNA synthesis across the full length of the transcripts with as little as 1 ng of total RNA, thus improving the coverage of our whole-transcriptome amplification independently of the 3’polyA tail [37]. We, then, barcoded, pooled and sequenced our eight RNA pull-down libraries on the Illumina HiSeq-2000 platform (see Methods). The resulting paired reads were computationally assembled and mapped onto the *C. elegans* WS190 genome. A summary of the results of this mapping is displayed in Table S1 in Additional file 1.

We obtained approximately 15 M unique reads per sample (approximately 130 million reads total). The number of genes mapped in each tissue was consistent between biological replicates, reflecting the robustness of our library preparation from tissue-specific RNA samples.

The overall number of genes and their ratio detected in each biological replicate, with the exception of our negative control, was comparable (about 1 versus 0.7) (Table S2 in Additional file 1), suggesting that our approach was consistent. In the intestine, we detected a much larger number of genes (7,355 genes) compared with that of pharynx (3,094 genes) and body muscle (3,604 genes) tissues.

Genes and their expression levels between biological replicates correlated well, with the exception of the *myo2p*::Δ*pab-1* control, further supporting the reproducibility of our approach and suggesting that PAT-Seq is specific and sensitive (Figure S1 in Additional file 1). A closer look into transcripts recovered with *myo2p*::Δ*pab-1* revealed an enrichment of ncRNAs and other common contaminants, reinforcing that this sample represented random non-specific RNA pulled-down during the immunoprecipitation step (Figure S1 in Additional file 1; Table S3 in Additional file 2). We have validated the tissue localization of selected tissue-specific genes identified with PAT-Seq by cloning their promoters and using them to drive expression of GFP *in vivo* (Figure S2 in Additional file 1).

**Figure 2:**
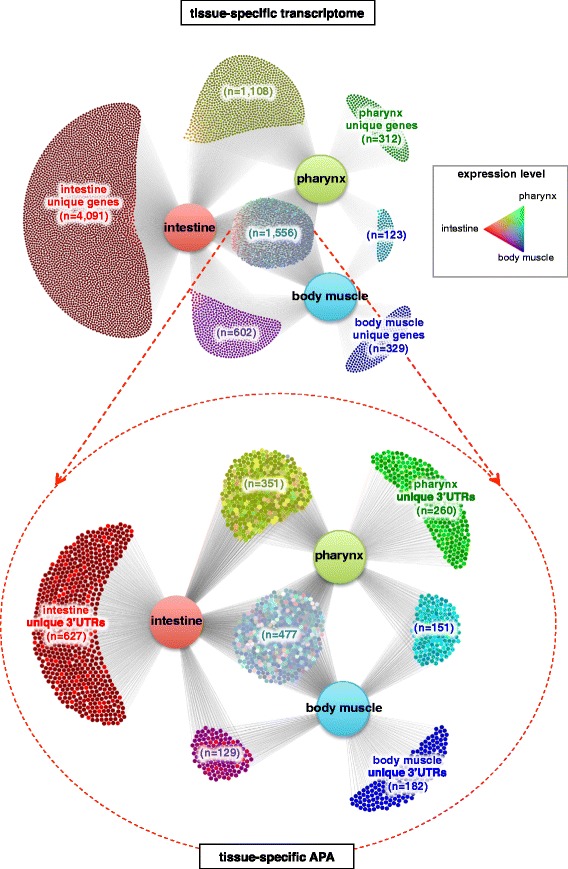
**Distribution of tissue** **-specific gene expression and alternative polyadenylation in intestine,** **pharynx and body muscle.** *Top panel*: Tissue-specific genes identified by PAT-Seq and the distribution of their expression levels between each tissue. A large pool of 4,091 genes is uniquely expressed in the intestine, while a smaller portion of 312 and 329 genes is expressed uniquely in the pharynx and body muscle, respectively. We have detected a common set of 1,556 genes expressed in all three tissues. Edges represent the presence of transcripts in each tissue, and color-coding indicates expression levels of genes in tissues (legend). *Bottom panel*: The 1,556 genes shared in all three tissues were further sorted based on the 3’UTR isoform and their expression levels. Approximately 25% to 30% of these genes use common 3’ends in these three tissues, while the remaining 70% use tissue-specific 3’UTR isoforms.

We detected a common core set of approximately 1,500 unique genes present in all three tissues. These transcripts include housekeeping genes, such as actin, histone genes, ribosomal proteins, genes involved in transcription, basal transcription factors, and genes involved in DNA maintenance and replication. A complete list of genes and isoforms detected in each tissue is shown as a diagram in Figure 2, top panel and comprehensively in Table S3 in Additional file 2.

### The intestine transcriptome is expansive, expressing over 30% of C. elegans mRNAs

The intestine is one of the largest tissues in *C. elegans*, composed of 20 large cells and a total of 30 to 34 nuclei, with a final 32-fold polyploidy in the adult worm. It is also one of the most functionally diverse tissues, participating in digestion, nutrient transport and storage, innate immunity, response to environmental toxins, defecation and dauer formation [38-40]. While the intestine transcriptome has been studied extensively [24,29,39], our results correlated with and significantly expanded these studies (Figure S3 in Additional file 1).

We detected a total of 7,355 expressed genes in this tissue (about 1/3 of the worm transcriptome), of which 4,091 genes and 4,634 spliced isoforms are uniquely expressed in the intestine, but not in either muscle tissue (Figure 2, top panel). The most abundant in this dataset were metabolic enzymes and nutrient transport genes, consistent with this tissue’s physiological function in digestion (Table S3 in Additional file 2). Among these intestine-only genes, we identified 212 unique transcripts that contain a DNA binding domain and were previously described as transcription factors [41,42] (Table S4 in Additional file 3). We speculate that these transcription factors may contribute to the gene regulatory network necessary for tissue identity and function. As expected, members of the GATA family are among the most abundant transcription factors. These factors bind ‘GATA’ elements on promoters and have been shown to regulate endoderm specification and all aspects of intestine development and function in *C. elegans* [43]. In addition, a significant portion (45%) of all transcription factors uniquely expressed in this tissue (96 out of 212) are members of the nuclear hormone receptor family. Many members of this class of transcription factors were also previously shown to regulate *C. elegans* metabolism [44]. We also detected a large pool of novel intestine-specific transcription factors with unknown roles that need to be further investigated (Table S4 in Additional file 3).

Recently, Pauli and colleagues coupled mRNA-tagging with DNA microarrays from L4-stage worms and identified 1,647 intestine transcripts [29] (Figure S3A in Additional file 1). Others produced a SAGE library of transcripts from dissected worm intestines from mutant adults, and detected a total of 5,623 intestine genes (Figure S3A in Additional file 1) [43]. A comparison of each of these datasets with ours identified 71% and 78% overlap with McGhee and Pauli datasets, respectively (Figure S3B in Additional file 1), suggesting that the increased sensitivity of PAT-Seq applied to study the intestine transcriptome of mixed stage worms expanded the core *C. elegans* intestine transcriptome.

Haenni and colleagues [24] recently optimized a procedure for extracting intact nuclei from *C. elegans* intestine, followed by fluorescence-activated cell sorting (FACS) and deep sequencing of mRNA, focusing on the 3’ end of the transcriptome. This approach allowed the authors to map 3,502 genes expressed in this tissue (Figure S3A in Additional file 1). We compared our intestine datasets with these results, downloading and remapping the raw reads from Haenni *et al*. using the same filtering criteria used in our datasets (see Methods). We found that 71% of genes were detected in both datasets, leaving 29% of genes present in the Haenni *et al*. dataset not present in ours (Figure S3C in Additional file 1). Despite these differences, the distribution of genes detected in both datasets plotted by expression values correlated (Figure S3D in Additional file 1). Our inability to detect 1/3 of the genes in Haenni *et al*. may be attributed to the fundamentally different techniques used in the preparation of our cytoplasmic, intestine-specific mRNAs using Pat-Seq versus nuclear cDNA prepared from isolated FACS sorted nuclei as in Haenni *et al*. (Figure S3E in Additional file 1). The difference in gene pools may have arisen because of the different cellular origin of the mRNAs analyzed by the two studies.

Finally, we have overlapped our datasets with McGhee *et al*. [42] and Haenni *et al*., the two intestine transcriptomes datasets obtained by sequencing, revealing a core set of shared 1,045 genes (Figure S3F in Additional file 1). These 1,045 genes represent a collection of high confidence intestine expressed genes, supported by at least three independent approaches, and will provide important insights in unraveling the genetic basis of intestine tissue identity in worms.

### Muscle transcriptomes are smaller but contain mostly unique gene pools

*C. elegans* possess only two large muscles: the pharynx and the body muscle. The pharynx, or foregut, is an important developmental model composed of eight layers of muscle, in addition to surrounding neural and epithelial tissue [8]. The muscle component of this tissue is composed of 20 cells that coordinate intake and physical crushing of the worm bacterial diet [45], subsequently facilitating raw nutrient transfer to the intestinal lumen for digestion. While the genetic factors controlling early development of the pharynx have been described in detail [46], individual cell-types of the pharynx are less characterized because genetic signatures belonging to these specific subgroups have not been extensively studied.

We detected 3,099 genes expressed in pharynx muscle (Table S3 in Additional file 2 and Figure 2, top panel). Among the top genes expressed were several myosin and actin isoforms, and pharynx-specific neurotransmitters, consistent with this tissue’s muscular identity. Importantly, we found only 312 unique genes with 338 spliced isoforms significantly expressed in this tissue (approximately 10% of the total dataset) (Figure 2, top panel, Table S3 in Additional file 2 and Table S5 in Additional file 4). Most of these genes have unknown function, with only 70 transcripts (22%) described in Wormbase so far [47]. The top genes of this list are collagen isoforms and motor protein genes (Table S3 in Additional file 2). Within this pool we also detected 13 pharynx-specific transcription factors. Their gene targets are mostly unknown (Table S4 in Additional file 3).

The body muscle tissue, defined as all non-pharyngeal muscle cells, is homologous to vertebrate skeletal muscle [48], and critical for locomotion, egg laying, defecation, and mating [49]. Several groups also studied the transcriptome of this tissue [26,27]. We detected 2,610 genes expressed in the body muscle. Similar to our pharynx dataset, within this pool we detected only 329 unique genes corresponding to 365 spliced isoforms (Figure 2, top panel, Table S3 in Additional file 2 and Table S5 in Additional file 4). The list of top ten genes in the body wall muscle dataset was also enriched for muscle-specific genes such as myosin and actin isoforms (Table S3 in Additional file 2). We also detected a unique gene pool, including previously identified genes, such as those in the calveolin family [50], and type IV collagen [51]. Out of predicted worm transcription factors, we identified 22 genes that are uniquely expressed in this tissue (Table S4 in Additional file 3), including *unc-120*, a SRF-like transcription factor essential for body wall muscle development, and *blmp-1*, a PRDM family member required for embryonic slow muscle fiber formation in vertebrates [48,52].

Using PAT-Seq we were able to detect many statistically significant isoform expression changes between intestine and muscle tissues (Figure S4 in Additional file 1 and Table S5 in Additional file 4).

### Tissue-specific promoters possess unique signatures

The compact nature of the promoter regions in the *C. elegans* genome provides us with a unique platform to examine tissue-specific elements in these regions and highlight signatures such as transcription factor binding sites. We first studied the sequence composition of these promoters, defined as the portion of genomic sequences from -500 to +100 from the TSS of protein coding genes [24]. We compared these sequences to promoters of random genes from the whole *C. elegans* transcriptome (28,122 genes from WS190) (Figure S5A in Additional file 1). We found that, contrary to higher metazoans, such as in humans where promoters are significantly enriched in cytosines and guanosines, *C. elegans* promoters are significantly enriched in adenosine and thymidine. These two nucleotides represent more than 66% of the total nucleotide composition in these promoter regions (*data not shown*). We also detected a strong T-rich region closer to the transcription start site of intestine genes, which perhaps is implicated in intestine-specific mechanisms of transcription initiation (Figure S5A in Additional file 1).

We then scanned these promoter regions for enriched elements uniquely present in this tissue, hoping to detect tissue-specific signatures. We calculated the frequency of all possible hexamers within the promoter regions of our intestine and random datasets, and then gathered the frequency of these elements into six bins consisting of 100 nucleotides each (Figure S5B in Additional file 1). Among the top hits, we detected a significant enrichment of many ‘GATA’ binding sites in these promoters (24% to 40% higher) (Figure S5B and S5C in Additional file 1).

We expanded these analyses in the muscle tissues and scanned promoter regions for enriched hexamers and known *trans*-acting factors (Table S6 in Additional file 1). Although we used unique gene datasets to search for tissue-specific motifs, the body muscle and pharynx shared several highly significant sequences, suggesting the existence of common core regulatory elements modified for pharynx and body muscle (Table S6 in Additional file 1).

Many eukaryotic promoters contain a ‘TATAA’ binding element used to recruit the transcription machinery to the transcription start site. When we extended this search in promoter regions in the worm genome (WS190) and in our three tissues, the frequency of the ‘TATAA’ box was approximately 37%, slightly higher than what is observed in human (24%) [53] (*data not shown*), suggesting that while not ubiquitous, the TATA box is still abundant in nematodes. A list of all significantly enriched motifs detected in intestine, pharynx and body muscle promoters is shown in Table S6 in Additional file 1.

### Tissue-specific 3’end formation events are unlikely driven by unique sequence signatures in intestine and muscle tissues

We next studied changes in PAS and APA in these datasets. APA is pervasive in *C. elegans* [5,6], but it is still unclear in which tissues these 3’UTR isoforms are expressed, how they are produced and their consequences. We employed an innovative library preparation method based on isothermal linear amplification of polyA+ RNA, which allowed us to bypass ligation-based approaches and precisely detect both the transcriptome and 3’UTRome of selected tissues profiled at the same time (see Methods). This method, named SPIA, produces continuous linear synthesis of ssDNA amplicons from a single RNA template, producing consistent read numbers through the transcriptome and minimizing internal mis-priming that could generate false 3’ends during the cDNA library preparation [37].

**Figure 3:**
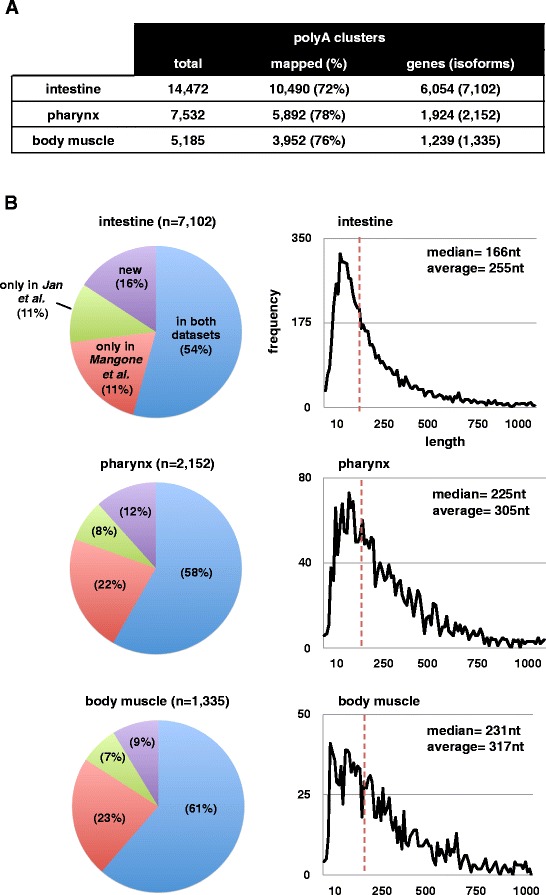
**3** **’UTR poly-** **A site mapping in tissue datasets.** We used the raw sequencing reads to map high-quality polyA sites onto the WS190 worm annotation and compared our results with two published *C. elegans* 3’UTRome datasets. **(A)** The number of polyA clusters mapped from polyA-containing sequencing reads (total), the portion of those that mapped to the WS190 worm genome annotation (mapped), the number of genes with polyA sites mapped (closest gene to the polyA cluster) and the number of isoforms resulting from distinct mapping of polyA clusters (isoforms). **(B)** The majority of mapped 3’UTR isoforms are supported by two published 3’UTRomes and almost 90% of them are supported by at least one dataset. *Left panel:* the percentage of isoforms mapped to either of two published 3’UTRomes (green and red), to both (blue), and those not present in either 3’UTRome dataset (purple). *Right panel:* the distribution of 3’UTR length for all 3’UTR isoforms found in each tissue dataset, along with the median (vertical dashed red line) and the average length.

Using this approach, we were able to build approximately 20,000 high-quality PAS clusters (72% to 78% of the total mapped PolyA clusters) that allowed us to map 3’UTR ends at single base resolution for approximately 6,000, 2,000, and 1,200 genes in our intestine and two muscle datasets, respectively (Figure 3A). Importantly, more than 80% of these mapped 3’UTR isoforms overlapped with previously described datasets [5,6] (Figure 3B), strongly suggesting that the vast majority of 3’UTRs detected using our approach are *bona fide* 3’ends of mRNAs.

We then studied the length of the 3’UTRs in these tissues and found that, on average, intestine genes possess shorter 3’UTRs, when compared to pharynx and body muscle genes (Figure 3B). In mammals, shorter 3’UTRs tend to escape post-transcriptional gene regulation and are more stable in comparison with longer mRNAs [54]. This activity has not yet been documented in worms, but presumably these short 3’UTRs could lead to an increase in protein translation in the *C. elegans* intestine to support its diverse physiological roles.

Next, we analyzed the PAS elements, which are sequences in 3’UTRs known to direct 3’end formation. We superimposed our tissue-specific datasets to the worm 3’UTRome from the modENCODE project [5] and extracted the PAS nucleotide composition (Figure S6 in Additional file 1). The canonical PAS element ‘AAUAAA’ in intestine and muscle tissues was approximately 10% more abundant than in the 3’UTRome overall (Figure S6 in Additional file 1). The PAS sequences containing one permutation of the canonical element were similar in all three tissues, while those containing two or more permutations were drastically reduced (Figure S6 in Additional file 1). PAS position within 3’ ends of mRNAs was similar in all three tissues (Figure S7A in Additional file 1).

We then studied the sequence conservation near the mRNA cleavage sites in genes present in each dataset, hoping to detect tissue-specific signatures (Figure S7B in Additional file 1). The nucleotide frequency in these regions was remarkably similar between tissues (Figure S7 in Additional file 1). We detected only a slight change in frequency of adenosines near the PAS site, which was specific to 3’UTRs expressed in the intestine (dashed box in Figure S7B in Additional file 1).

Taken together, our results suggest that these sequences may not contain elements important in tissue-specific 3’ end formation or that such elements are further downstream of the cleavage site and not detected by our analysis.

### Alternative polyadenylation is pervasive in intestine and muscle tissues

**Figure 4:**
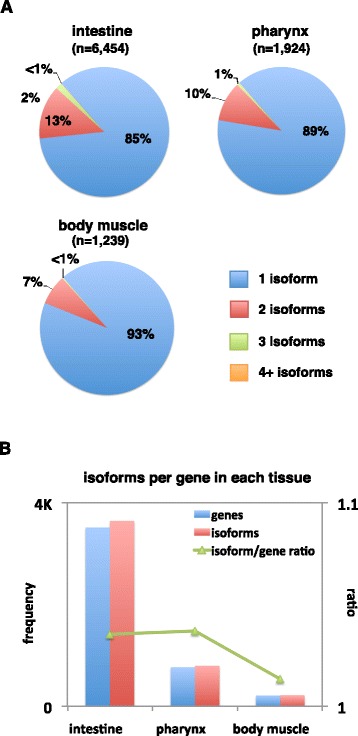
**Abundance of APA in** ***C*** **.** ***elegans*** **tissues.** A finalized list of genes with mapped 3’UTR isoforms was generated for each tissue and used to compare the abundance of 3’UTR isoforms between tissues. **(A)** Proportion of genes subject to alternative polyadenylation in each tissue. The intestine expressed significantly more genes containing more than one 3’UTR isoform, while the muscle tissues expressed similar proportions of genes with more than one 3’UTR isoform. **(B)** The average number of 3’UTR isoforms detected for each gene/tissue. The number of genes and isoforms (frequency) are displayed in each column (left x-axis). We calculated and displayed the change in 3’UTR isoform to gene ratio (right x-axis) between each tissue (green trend line). We detected slightly more APA in the intestine and pharynx, when compared with the body muscle tissue.

The two available worm 3’UTRome datasets estimate that approximately 46% of *C. elegans* genes use APA [5,6]. APA is coordinated through development, where proximal 3’UTRs are expressed in earlier developmental stages and distal are expressed more frequently in later developmental stages [5]. However, the extent to which APA is coordinated between *C. elegans* tissues and how it may participate to establish cell identity has not yet been addressed. We employed a normalization method to select for higher confidence 3’UTR isoform switching events based on the ratio between PAS coverage (see Methods). Intestine tissue has a larger pool of genes with two or more 3’UTR isoforms (twice as many genes as in muscle tissues), while muscle tissues mostly use single 3’UTR isoforms (Figure 4).

**Figure 5:**
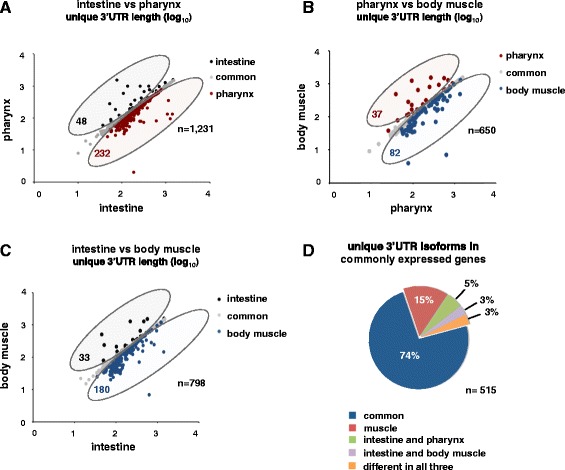
**APA is pervasive between** ***C*** **.** ***elegans*** **tissues.** We have followed 3’UTR length changes in genes with only one 3’UTR isoform between intestine, pharynx and body muscle tissues. Length comparison between the same genes expressed between **(A)** intestine and pharynx, **(B)** pharynx and body muscle and **(C)** intestine and body muscle tissues. Shaded circles represent those genes expressed with proximal 3’UTR isoforms in the intestine (black), pharynx (red) or body muscle (blue), where the distal isoform was detected in the other corresponding tissue in each graph. Genes with 3’UTR isoforms that were the same length between each tissue are represented in grey as noted in the legend. **(D)** Distribution of unique 3’UTR isoforms for genes detected in all three tissues. The majority of these 3’UTRs are common in all three tissues (blue). Genes with a 3’UTR isoform in the intestine distinct from muscle tissues are also abundant (muscle shared). Only 2% of these genes express different 3’UTR isoforms between all three tissues (distinct).

We reasoned that if APA is a tissue-specific event, we would be able to detect it by following the dynamics of 3’end formation in genes with one 3’UTR isoform detected in each tissue. Indeed, we found that 18% to 26% of those genes switched 3’UTR isoform in a tissue-specific manner (Figure 5); a comprehensive list of these genes is displayed in Table S7 in Additional file 5. Interestingly, intestine genes more often used distal PAS sites, while both muscle tissues used proximal PAS sites. When we focused this analysis comparing 3’UTR switches in genes expressed in all three tissues, we found that approximately 25% of these genes use APA in a tissue-specific manner (Figure 5D), suggesting that tissue-specific APA in worms is abundant.

### Tissue-specific 3’UTR isoforms are enriched with predicted and experimentally validated miRNA targets

**Figure 6:**
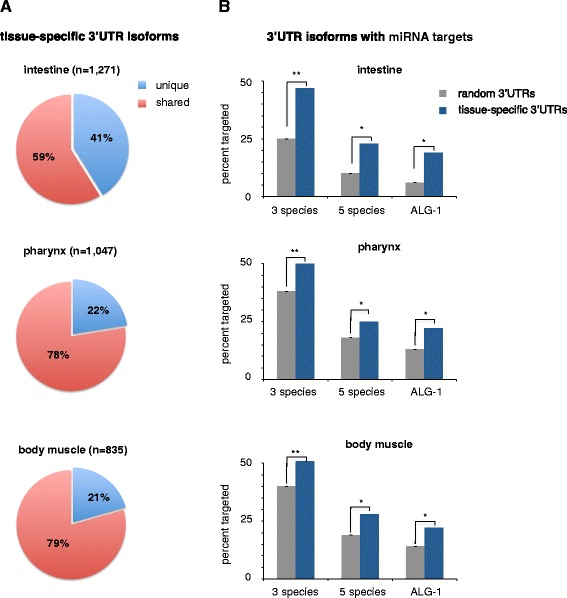
**Analysis of tissue-specific 3**’**UTR isoforms.** We calculated the proportions of genes in each tissue that have tissue-specific 3’UTR isoforms and how many of these 3’UTRs have predicted microRNA targets. **(A)** Charts displaying the proportion of genes containing tissue-specific 3’UTR isoforms (blue). The intestine expresses approximately two times as many tissue-specific 3’UTR isoforms as muscle tissues. **(B)** We compared the proportion of microRNA targeted genes with tissue-specific 3’UTRs (blue) to the same number of randomly selected genes (grey) in each tissue. Significantly more genes with tissue-specific 3’UTR isoforms have microRNA targets. microRNA targets were predicted using PicTar Software, using three species and five species conservation criteria, and from ALG-1 pull-down experiments (Zisoulis *et al*. 2010 [32]). *P-value <0.05, **P < 0.01, based on two-tailed Student’s *t*-test.

We searched within our three datasets for commonly expressed genes with tissue-specific 3’UTR isoforms (Figure 6A). While muscle tissues had a similar set of genes with tissue specific 3’UTR isoforms, the intestine tissue had, on average, twice the amount (Figure 6A), suggesting that the *C. elegans* intestine uses more APA than the two muscle tissues.

Since we were able to detect widespread tissue-specific APA, we were interested to study if the genes that use tissue-specific APA were enriched with miRNAs targets, and perhaps use APA to escape their regulation. We searched in each tissue for genes with tissue-specific 3’UTR isoforms that have bioinformatically predicted microRNA targets (Figure 6B). Remarkably, genes with tissue-specific 3’UTR isoforms were enriched with miRNA targets using both a three-species (approximately 49%) and a five-species (approximately 25%) conservation filter (Figure 6B). This is significantly more abundant than the average number of total genes expressed in each tissue having miRNA targets using the same criteria (Figure 6B).

miRNA prediction software produces significantly high false and negative hits that cannot be used to properly assign targets [55]. When we instead compared our dataset to *in vivo* miRNA target footprint data from past studies [32], we found a similar enrichment, with an average of 21% of genes with tissue-specific 3’UTR isoforms containing validated targets (Figure 6B).

In conclusion, these data suggest that miRNA targets are much more abundant in ubiquitously expressed genes with tissue-specific 3’UTR isoforms than in genes without APA, strongly linking APA to miRNA targeting and post-transcriptional gene regulation. Our data support a model whereby the 3’UTRs of genes with APA are regulated in a tissue-specific manner in order to evade or participate in microRNA targeting.

### APAome.org, a tissue transcriptome resource for C. elegans biology

We have made our data publically accessible through our APAome website [33]. The APAome includes our tissue-specific datasets, as well as other important worm 3’UTR datasets [5,6,24], allowing the community to have a comprehensive view of APA and 3’UTR biology in worms. The APAome database [33] provides detailed information on 3’UTR isoforms for all protein-coding mRNAs present in Wormbase [47], novel PicTar [5] and TargetScan [6] predictions, and includes annotations extracted from other databases as well as new annotations generated by others.

## Discussion

In this study, we have coupled mRNA-tagging with high-throughput sequencing in a novel technique that we called PAT-Seq, and used it to perform an integrative analysis of the mRNA transcriptome of *C. elegans* intestine, pharynx and body wall muscle tissues. We have studied their transcriptome and 3’UTRome at an unprecedented resolution. In addition, since these three libraries were prepared using the same approach, we were able to directly compare the changes in gene expression and the gene content across these three somatic tissues, without extrapolating data from other studies. Our approach is an improvement over past methodologies [34,35], allowing the identification of tissue specific mRNAs at higher resolution.

### PAT-Seq highlights C. elegans intestine and muscle transcriptome dynamics

We found that the intestine transcriptome is significantly larger than in muscle tissues, possibly to support its especially diverse physiological roles. Intestinal cells are much larger and are increasingly polyploid throughout larval development, with more transcriptional capacity compared to the smaller, diploid muscle cells [56]. Although there are no other comparative data in worms, recent genome-wide transcriptome analyses in the human intestine track support our findings, showing that more than 75% of all protein-coding genes are expressed in this tissue [57]. The twenty large, intestinal cells may require a large pool of distinct genes to carry out functions specific to their anatomical location, since altogether these cells span from the pharynx to the posterior of the animal and the intestine is one of the largest tissues in worms.

Overall, intestinal tissue is more different in gene composition than the two profiled muscle tissues. In intestine, the most abundant genes detected are common metabolic enzymes, such as *fat-1*, *pmt-1*, *asp-1,* and others, which were also detected in other available intestine-enriched datasets [24]. Importantly, genes and isoforms detected in our intestine dataset correlated with, and significantly expanded, previously described worm tissue-specific datasets [24,29,43]. In the pharynx and body muscle, the top genes detected were myosin genes, actin isoforms and other genes. We also detected a large number of tissue-specific alternative splicing events and fold change differences in gene expression for genes in common between tissues.

Gene expression changes could be caused by stage-specific enrichments in our pull-down experiments. We have prepared our tissue specific RNAs using a well-established protocol that allows the growth of worms in liquid media [58]. This protocol is known to provide an even representation of each worm developmental stage. Since all our samples were prepared using the same protocol, it is unlikely that a given sample is biased towards a specific developmental stage. Importantly, our intestine dataset overlaps consistently with recently published studies [24,29,43], suggesting that if there is a general bias in all three tissue-specific datasets, it is very low.

Our study detected many tissue specific genes in intestine and muscles that were previously reported by others in the same tissues (Wormbase). We have also validated a selected portion of these hits using a GFP reporter approach [see Additional file 1: Figure S2]. We think that although very sensitive and reproducible, our PAT-Seq approach may introduce some noise at the lower end of detection. Further experiments may need to be performed to validate the tissue specific localization of these low expressed genes.

### C. elegans promoters are AT-rich and contain tissue-specific motifs

Our promoter analysis showed that worm promoters are AT-rich. This result is consistent with what others have found in worm genomic regions [59], *Drosophila* [60], and *Xenopus* [61], and very different from what is observed in mammalian promoters, which are GC-rich [62]. GC-rich regions in promoters increase genomic thermostability [63], provide more binding motifs for transcriptional activators [61] and support promoter gene silencing through DNA methylation at GC islands. The AT-rich nature of *C. elegans* promoters was previously observed in *C. elegans* genes expressed in the germline [64], and perhaps reflects a simpler model of transcription initiation with reduced regulation.

We also demonstrate that this approach can effectively identify previously reported and novel sequence elements in tissue-specific gene datasets. As a *proof-of-concept*, we detected GATA transcription factors known to be critical for all aspects of *C. elegans* intestine development and adult function in our intestine transcriptome [24,29,65], and detected potential GATA sites present at a higher frequency in the promoters of intestine-specific genes. Importantly, our study highlighted the presence of many novel enriched sequence motifs, many of which have not been described yet in the literature. While we were able to predict several transcription factors that could recognize these motifs, we still do not know if they are indeed functional, and further *in vivo* studies need to be performed to further characterize their role.

### SPIA library preparation increases yield and robustness of polyA sequencing

We have sequenced our tissue-specific libraries using a proprietary library preparation method, named Single Primer Isothermal Amplification (SPIA), which is ideal for use with mRNA-tagging, since the RNA yield from this approach is typically low [37,66]. Unlike recently developed methods used to map 3’UTRs, such as 3P-Seq [6] and FANS/3’-end-seq [24], SPIA generates cDNA libraries that cover the entire transcript, allowing for more extensive downstream transcriptome analysis within the same experiment, such as coupling gene isoform mapping with the study of 3’UTR dynamics. Since there is no amplification step, SPIA significantly minimizes internal mis-priming that could generate false 3’ends during the cDNA preparation [37]. It is important to note that PAT-Seq relies on the binding affinity of *pab-1* to polyA tails of mature mRNAs, which are known to change in length in eukaryotic genes [67]. This in turn can create difficulties in the quantification of gene expression levels of libraries prepared with this technology. Although this is an inherent problem in all RNA-IP based approaches, our datasets correlated with previously published studies that did not use a PABPC-based approach [see Additional file 1: Figure S3] [24].

Using SPIA, we were able to build approximately 20,000 high-quality PAS clusters that allowed us to map 3’UTR ends at unprecedented resolution for approximately 6,000, 2,000, and 1,200 genes in intestine, pharynx and body muscle datasets, respectively. Importantly, we found that more than 80% of these 3’UTR isoforms overlap with previously described datasets [5,6], strongly suggesting that the vast majority of 3’UTRs detected by our approach are *bona fide* 3’ends of mRNAs.

### Approximately 18% to 26% of genes use tissue-specific APA

We show that approximately 18% to 26% of total genes detected in intestine and muscle tissues used tissue-specific APA. Intestine genes seemed to favor *proximal-to-distal* PAS switches, leading to longer 3’UTR isoforms, while both muscle tissues alternated 3’UTR isoforms at a similar rate. Previous studies of 3’UTR datasets reported that as many as 46% of worm genes use APA across multiple tissues and developmental stages [5,6]. This apparent discordance with our findings indicates that a large majority of genes in worms use only one 3’UTR isoform in a given tissue, suggesting that APA is indeed an important mechanism used by cells to regulate gene expression at the tissue-specific level.

Importantly, our work identified an overall high number of novel 3’UTR isoforms that were not present in past analyses [5,6]. This pool spans from 9% to 16%, depending of the tissue examined (Figure 3B). Previous work reported that the worm 3’UTRome is not saturated and other novel 3’UTR isoforms may be present [5]. Interestingly, the majority of the 3’UTR isoforms within this pool are tissue-specific (82% in intestine and 58% in the muscle), suggesting that perhaps these 3’UTR isoforms are also rare and were not identified in earlier studies because of the limit of sensitivity of their mixed-tissue, transcriptome-wide approaches [5,6].

Our analysis uncovered significant APA in worm tissues, but we could not identify upstream tissue-specific elements involved in 3’end formation, suggesting that in worms, other accessory tissue-specific factors [68] or their dosage [19] may play a role instead [5,6].

### Tissue-specific 3’UTR isoforms are linked to microRNA regulation

Past dogma that the protein and the transcription levels in cells are directly proportional is not accurate anymore [69,70]. Thanks to the introduction of novel high-throughput technologies, it is now clear that there is not a direct correlation between the transcriptomes and the proteomes of cells or tissues. Instead, miRNAs, together with other ncRNAs and RNA binding proteins, play key roles in modulating the final gene output on its way to protein expression [14]. This modulation, when combined with the abundance of APA detected in this study, suggests a more complex picture, where there are not only negative regulatory networks through miRNAs, but also novel unexplored positive regulatory networks operated though APA. These positive networks are driven by genes that switch between 3’UTR isoforms to escape miRNA targeting, allowing their expression. In this view, both miRNAs and APA can, in principle, dramatically reshape gene expression output, implying they both play key roles in the establishment and maintenance of cell and tissue identity.

In this study we found that genes with tissue-specific 3’UTR isoforms are enriched in microRNA targets using both a three-species (approximately 49%) and a five-species (approximately 25%) conservation criteria. This was significantly more abundant than what we saw in randomly selected 3’UTR isoforms using a three-species (25% to 40%) and a five-species (10% to 19%) conservation criteria in each tissue. We also found a similar enrichment comparing our dataset to the experimentally validated ALG-1 footprints [32]. Our results in three worm somatic tissues link miRNA regulation to APA, showing that microRNA targets are much more abundant in ubiquitously expressed genes with tissue-specific 3’UTR isoforms than in genes that do not use APA.

Recently, microRNA populations from intestine and body muscle tissues were isolated using an RNA-IP strategy, providing a tissue-level atlas of microRNA expression [71]. Unfortunately, while this study suggests that miRNAs are also pervasive in worm tissues, it is still unclear which genes they target, and further experiments have to be performed to highlight these regulatory networks.

### APAome.org: a resource for 3’UTR biology

We have compiled our tissue-specific transcriptomes into a useful online resource for scientists interested in 3’UTR biology and APA. The APAome.org site uses an Apache web server and several custom-made Perl scripts that query a dedicated MySQL database. It is currently hosted in the Biodesign Institute at Arizona State University, and offers a simple and well-integrated interactive user interface to query gene records and 3’UTR isoform data, giving access to a dedicated gBrowse installation specifically designed to study APA in worms. This database displays tracks for each tissue transcriptome, including tissue-specific APA, as well as curated 3’UTR data from previously published studies [5,6,24].

## Conclusions

Here we present the first comparative analysis of the transcriptome, the 3’UTRome, and the promoter diversity of three large somatic tissues of the soil nematode *C. elegans*. We found abundant tissue-specific gene expression changes that correlated with the presence of distinct promoter signatures between *C. elegans* intestine and muscle tissues, and defined thousands of tissue-specific splice isoforms.

We have discovered that tissue-specific APA is pervasive in nematodes, and that 3’UTR isoform changes correlated with gain or loss of miRNA target elements, suggesting a role for APA in modulating tissue-specific post-transcriptional gene regulation.

## Methods

### Plasmids and molecular cloning

The PolyA-Pull plasmid was constructed adapting the Gateway pDONR221 (Invitrogen, Carlsbad, CA) as follows. The *pab-1* gene was amplified from N2 genomic DNA using a forward specific primer containing a SacII site, and a reverse specific primer containing BamHI and EcoRI sites (Table S8 in Additional file 6). The amplicon was then ligated *in-frame* with GFP (Marco Mangone, *unpublished*) using T4 DNA Ligase (NEB, Ipswich, MA, USA) and SacII and EcoRI sites. The 3 × FLAG epitope DNA sequence was obtained from the DNASU Plasmid Repository [72] (DNASU clone ID: HsCD00298297) and extracted using PCR amplification using a forward primer containing a BamHI site and reverse primer containing an EcoRI site. The amplicon was then ligated into pDONR221 (Invitrogen) downstream and *in-frame* with the *pab-1* gene using T4 DNA Ligase (NEB). The Δ*pab-1*-Pull plasmid (GFP::Δ*pab-1*::3 × FLAG), which does not contain the *pab-1* sequence and cannot bind polyA+ mRNAs, was prepared from the PolyA-Pull plasmid using the Stratagene QuikChange® Site-Directed Mutagenesis Kit following the manufacturer’s guidelines (Stratagene, La Jolla, CA, USA) (Table S8 in Additional file 6). The 3’UTR of the *unc-54* gene, cloned in Gateway pDONR P2R-P3 entry vectors [5], was used as an unspecific 3’UTR in all of the destination vectors in this study. The tissue-specific promoters were selected as the genomic sequence of DNA upstream of their transcription start site, up to 2 kb. We have designed the primers using the University of California, Santa Cruz (UCSC) Genome Browser and cloned the resultant amplicons from N2 genomic DNA into the Gateway™ pDONR P4-P1R entry plasmid (Invitrogen) (Table S8 in Additional file 6). We used Multisite recombination reactions (LR Clonase plus II, Invitrogen) to join the tissue specific promoters, the PolyA-Pull vector, and the *unc-54* 3’UTRs into the Gateway Compatible MosSCI destination plasmid pCFJ150 [36] (Addgene plasmid #19329), and used these vectors for the preparation of the transgenic strains.

### Nematode strains and preparation of transgenic animals

*Wild-type* strain N2 worms were obtained from the *Caenorhabditis* Genetics Center (CGC) (University of Minnesota), which is funded by NIH Office of Research Infrastructure Programs (P40 OD010440). Worms of strain EG4322 (to prepare MosSCI transgenics) were maintained at 16°C on HB101 containing nematode growth media (NGM) agar plates prior to microinjection [2]. Stable transgenic worm strains were prepared using the MosSCI technology as described [3]. Microinjection mixes consisting of pJL43.1(50 ng/μl), pCFJ90(1 ng/μl), pGH8(10 ng/μl), pCFJ104(5 ng/μl), and pCFJ150::TissuePromoter::GFP::*pab-1*::3 × Flag::*unc-54* (25 ng/μl) were microinjected into worm strain EG4322 (ttTi5605; *unc-119(ed9) III*), each of which was kindly provided by Priscilla Van Wynsberghe (Colgate University, Hamilton, NY, USA). Microinjection was carried out using a Leica DMI3000B microscope as described previously [36,74]. Injected worms were plated on NGM growth media plates containing OP51 bacteria, and plates containing *unc-119* rescued (mobile) worms were chunked onto four new NGM plates and left to starve for at least 30 days at 25°C. Single dauer worms were plated onto small NGM plates, propagated for approximately two weeks, and verified for GFP expression using a Leica DMI3000B. DIC and fluorescent images were captured using a Leica DFC345FX mounted camera.

### Worm gDNA extraction and MosSCI insertion verification

Genomic DNA was phenol-chloroform extracted from one full 60 mm NGM plate from each transgenic worm strain, precipitated with sodium acetate and washed in ethanol. To confirm the MosSCI integration of transgenes into the ttTi5605 intergenic region, we performed PCR using Standard Taq Polymerase (NEB) using a forward primer annealing outside of the homologous flanking region (5’- CCTCTGAACTGGTACCTCA -3’) and a reverse primer annealing within the *unc-119* rescue cassette (5’- GGAAGAAGGAAAAGAGTGTGG -3’), both of which were provided by Priscilla Van Wynsberghe (Colgate University).

### Western blotting

Western blotting for detection of the GFP::PAB-1::3 × FLAG fusion protein in transgenic worms was carried out as follows. One full 60 mm NGM plate of worms was washed with M9 media into a 1.5 ml centrifuge tube and pelleted at 1,500 rpm. Worms were washed 2× in PBS buffer and then resuspended in an equal volume of sample buffer (125 mM Tris-Cl (pH 6.8) 4% SDS, 20% glycerol, 0.5% bromophenol blue) supplemented with 10% beta-mercaptoethanol and boiled at 95°C for five minutes. The reaction was spun down and 15 μl of supernatant was run at 200 V on a 4% to 15% Tris-Glycine Criterion™ precast polyacrylamide gel (Bio-Rad, Hercules, CA, USA) for 36 minutes. Electrophoretically separated proteins were transferred to an Amersham Hybond™-P blotting membrane (GE Healthcare, Little Chalfont, UK) using a Trans-Blot SemiDry Transfer Cell (Bio-Rad) at 23 V for one hour. The membrane was blocked in blocking buffer (5% milk in PBS with 0.01% TWEEN-20) for one hour at room temperature followed by overnight incubation with ANTI-FLAG® antibody produced in rabbit (Sigma-Aldrich, St. Louis, MO, USA). Following incubation, the membrane was washed 3× in blocking buffer and then incubated with a 1:1000 dilution of anti-Rabbit immunoglobulin G (IgG) horseradish peroxidase (HRP)-linked secondary antibody (Cell Signaling, Danvers, MA #7074S) in blocking buffer for one hour. The membrane was finally washed 4× in PBST (1xPBS, 0.01% TWEEN-20) and then reacted with SuperSignal ELISA Femto Maximum HRP Substrate (Thermo Scientific, Rockford, IL, USA), followed by imaging with a FluorChem FC2 Imager (Alpha Innotech, San Leandro, CA, USA).

### RNA immunoprecipitation

The mRNA tagging technique was adapted from past studies [32,34]. Mixed-stage liquid worm cultures were grown as described [58] at 20°C. Approximately 10^6^*pab-1*::3 × FLAG transgenic worms were harvested from liquid culture after three to four days, crosslinked for one hour in 0.5% paraformaldehyde in M9 solution, and flash frozen in ethanol-dry ice bath. Frozen pellets were crushed using a mortar and pestle in liquid nitrogen and the resulting frozen powder was transferred directly into lysis buffer (150 mM NaCl, 25 mM HEPES, pH 7.5, 0.2 mM dithiothreitol (DTT), 10% glycerol, 0.0625% RNAsin, 1% Triton X-100), described in [75]. Total RNA was extracted from worm lysates using Trizol® Reagent (Life Technologies, Carlsbad, CA, USA) and precipitated with isopropanol. An amount of lysate corresponding to 90 μg of total RNA was added to 100 μl of Anti-FLAG® M2 Magnetic Beads (Sigma-Aldrich) and incubated overnight at 4°C. Each reaction was washed 3× in 200 μl TBS and then 3× in 200 μl Proteinase-K buffer with 1,000 RPM mixing. Proteinase-K (4 mg/ml) was added to the beads and incubated at 37°C for 30 minutes with 1,000 RPM mixing. A total of 7 M urea was added to the beads and incubated at 37°C at 1,000 RPM before RNA was extracted with Trizol® Reagent and precipitated with isopropanol and GlycoBlue (Ambion, Austin, TX, USA). Precipitated RNA was treated with DNAse I (NEB) for ten minutes and extracted again with Trizol® Reagent and isopropanol. RNA was resuspended in nuclease-free water and quantified using a Nanodrop® 2000c spectrophotometer (Thermo-Fisher Scientific, Waltham, MA, USA).

### RT-PCR and 3’RACE reactions

Precipitated RNA (50 ng) was reverse transcribed with a NVdT_(23)_ primer using SuperScript Reverse Transcriptase III (Thermo-Fisher Scientific). Three microliters of the reverse transcription reaction was used in each PCR reaction using Standard Taq Polymerase (NEB) and primers specific to the 3’ end of each cDNA ORF (Table S8 in Additional file 6) or 3’UTR, as was the case for *unc-54* (forward primer sequence was extracted from previous publications [5]).

### cDNA library preparation and sequencing

The eight cDNA libraries were prepared using at least 50 ng of total RNA extracted from different tissues. We used the IntegenX’s (Pleasanton, CA) automated Apollo 324 robotic preparation system to reverse transcribe RNA into cDNA and for DNA library preparation. The cDNA synthesis was performed using a SPIA (Single Primer Isothermal Amplification) kit (IntegenX and NuGEN, San Carlos, CA) [37]. Once the cDNA was generated, we assessed the quantity of the cDNA libraries using the Nanodrop instrument (Thermo-Fisher). The cDNA shearing was performed on a Covaris S220 system (Covaris, Woburn, MA). After the cDNA was sheared to approximately 300 base pair fragments, the Nanodrop instrument (Thermo-Fisher) was used again to quantify the cDNAs in order to calculate the appropriate amount of cDNA necessary for library construction. Tissue-specific barcodes were then added to each cDNA library. The resultant eight tissue-specific libraries were then pooled and sequenced using the HiSeq platform (Illumina, San Diego, CA) with a 2 × 100 bp HiSeq run.

### Bioinformatics analysis of RNA-Seq data

#### Raw reads mapping

Paired raw reads were demultiplexed by their unique tissue-specific barcodes and converted individually to FASTQ files by the CASAVA software (Illumina). Unique datasets were then mapped to the *C. elegans* gene model WS190 using the Burrows-Wheeler Aligner software (BWA) [76] with default parameters. A summary of the results produced by this approach is shown in Table S1 in Additional file 1. Mapped reads were further converted into a bam format and sorted using SAMtools software run with generic parameters [77].

#### Cufflinks/Cuffdiff analysis

Expression levels of individual transcripts were estimated from the bam files by using Cufflinks software [79]. The fragment per kilobase per million base (FPKM) number was used to indicate the gene expression levels, and an FPKM value ≥1 was used as a threshold across all tissues profiled for defining expressed genes. The gene expression levels obtained in each tissue dataset were compared pairwise with other tissues using the Cuffdiff algorithm [78]. The Cuffdiff algorithm detected 389 isoforms shared between pharynx and intestine, 286 between body muscle and intestine, and 175 between the two muscle tissues (*P*-value <0.05). The results are shown in Figure S4 in Additional file 1. Cufflinks was unable to assign an FPKM value for eight genes in our intestine dataset (*vit-5*, *rpl-24.1*, ZK484.1, *hmg-1.1*, *rps-12*, Y24D9A.8, *rps-8* and *rpl-7A*). These genes were omitted in this study. The differential mRNA isoform analysis was performed with the CummeRbund package [79] using the output produced by the Cuffdiff algorithm. This analysis aimed to identify genes that change in expression level between tissues from large datasets. The data are displayed in Figure S4 as a plot. We have detected between 175 and 389 tissue specific isoforms that have significantly different expression levels between two tissues. These datasets are available as Additional file 4. Tissue-specific unique genes were assigned if they have an FPKM ≥1. Genes with an FPKM <1 were ignored in our analysis.

### Comparison with other intestine datasets

A list of 3,502 genes present in the original Haenni *et al*. dataset was obtained from the supplementary materials section from the publisher, and used for our analyses. In addition, we downloaded from GEO and re-mapped the original raw ‘sorted’ BAM file used in the Haenni *et al*. manuscript, using BWA [76] and Cufflinks [78] and standard parameters to the WS190 gene model. This mapping effort produced 5,840 clusters mapped to 3’UTRs of known genes with a FPKM ≥1. This list was labeled ‘Haenni *et al.,* re-mapped’ and used for our analysis. The list of genes detected by Pauli *et al*. and by McGhee *et al*. was obtained from the supplementary materials accompanying their respective manuscripts [29,43].

### Gene expression localization and validation

We cloned the promoter region of eight randomly chosen genes, designing genome-specific primers that selectively amplify promoter regions spanning from -2,000 nt to WS190-annotated start codon of the gene of interest. The results are shown in Figure S2 in Additional file 1. The genes chosen for this analysis were C25A1.5, *nac-3p*, *tmd-2p*, *fat-2p*, *nas-1p*, *let-756p* and *lin-3p*. These forward and reverse primers contain Gateway-compatible sequences to allow the cloning of the resulting promoter regions in Gateway-compatible entry vectors (Table S8 in Additional file 6). We used the GFP containing plasmid PolyA-Pull to drive GFP expression using these promoters. Each promoter was introduced at the 5’end of a MosSCI-compatible PolyA-Pull fused to the *unc-54* 3’UTR within the MosSCI-compatible destination vector pCFJ150 [80] using multisite Gateway recombination technology (Invitrogen). The finalized constructs were microinjected into young adult worms. At least two independent biological replicates per construct were screened for GFP expression. For each tissue, we defined a putative expression index, proportional to the FPKM values obtained for each gene in each tissue (* = FPKM <100, **, FPKM = 100 to 200, *** FPKM >200).

### PolyA cluster preparation and polyA mapping

To map polyA-sites to WS190 worm annotations, raw sequence reads were filtered using custom made Perl scripts. We extracted reads containing ≥30 consecutive adenine nucleotides at their 3’end. We obtained 14,472 total reads from intestine, 7,532 for pharynx, and 5,185 for body muscle (Figure 3A). The polyA elements were then removed and the reads were converted to FASTA format and aligned to the WS190 annotation using the Burrows-Wheeler Aligner [76] with standard parameters. Reads mapping to genomic regions containing ≥65% adenosines in either direction and/or with less than 18 consecutively mapped nucleotides were discarded. The reads produced approximately 27,000 high-quality PAS clusters mapped through the *C. elegans* genome. Each of these clusters was then bioinformatically attached to the closest gene within a 1,600 nt range in the same orientation. To increase the stringency of our analysis, we ignored clusters with <5% of the total number of polyA reads detected for a given gene, and PAS clusters that mapped genomic regions with >40% adenosines, to eliminate as much background as possible. Each cluster had a median length of approximately 70 nt with approximately 5 × coverage, and mapped 3’UTRs of genes detected in the corresponding tissue with a FPKM ≥1.

### PAS analysis

Mapped polyA sites were compared with Mangone *et al*. and Jan *et al*. to map common 3’UTR isoforms between these datasets. We assigned common PolyA sites if the overlap was between + -10 nt. PAS usage in Figure S6 in Additional file 1 was calculated as in Mangone *et al*. [5]. PAS position and PAS nucleotide composition for 3’UTR isoforms in each dataset was extracted from Mangone *et al*. and used for the analysis in Figure S6 in Additional file 1.

### PAS nucleotide frequency

We have bioinformatically extracted 70 nt sequences between -50 and +20 from the cleavage site of all 3’UTR isoforms detected in each tissue, and used these sequences to plot the nucleotide frequency.

### Promoter analysis

We have used custom Perl scripts to bioinformatically extract 600 nt from -500 to +100 from genomic regions of genes in WS190, in our intestine, pharynx, and body muscle datasets. We then calculated and displayed the nucleotide frequency in the graph shown in Figure S5A in Additional file 1. This approach was used in the past by others to study promoter regions [24]. The analysis in Figure S5B and S5C in Additional file 1 was performed binning these 600 nt-long promoter regions in 100 nt bins using custom Perl scripts (six bins total), and then calculating the frequency of all possible hexamer combinations in each bin. As a control, we have extracted genomic regions from a random set of genes. Each random dataset used in our analysis was composed of the same number of genes detected in each corresponding tissue. We then used custom Perl scripts to bin these regions in and search for enriched hexamers within each of these bins. Approximately 70% of worm genes are trans-spliced at their 5’ends, making it challenging to precisely identify worm promoter regions (Wormbook). The analysis in Additional file 1: Figure S5 Panel C excluded promoter regions of genes present in operons.

### Motif identification with MEME

Promoter regions from each tissue were subjected to analysis for enriched motifs using the MEME Suite [81]. We used the DREME tool to search for enriched short motifs (up to eight bases) in the tissue-specific promoter datasets used in our promoter analysis and performed a discriminative motif discovery search using different tissue datasets as negative controls. We then overlapped the motifs detected with DREME with the high quality transcription factor binding profile database JASPAR using the human and the worm datasets (version 2014) [82]. The results are shown in Table S6 in Additional file 1.

### Transcription factor search analysis

We searched our tissue specific datasets for the presence of known transcription factors present in the wTF2.0 database [41] and compared the results with Haerty *et al*. [42]. The results of this analysis are shown in Table S4 in Additional file 3.

### Gene expression network visualization with Cytoscape

Tissue-specific genes, isoforms, and APAs were extracted from the data tables, reconfigured as a binary interaction format with three tissue types and visualized as networks using Cytoscape v3.1 [83]. The FPKM values in the tissues were log2-transformed, converted to RGB color codes and used to display relative expression levels among three tissues.

## Additional files

Additional file 1: Figure S1. PAT-Seq sequencing results. **Figure S2.** Validation of tissue-specific genes detected by PAT-Seq. **Figure S3.** Comparative analysis with other available intestine-enriched datasets. **Figure S4.** Differential mRNA isoform expression analysis. **Figure S5.** Sequence analysis of promoter regions for intestine expressed genes. **Figure S6.** Analysis of PAS usage between tissues. **Figure S7.** PAS location and sequence requirement. **Table S1.** PAT-Seq raw sequencing data. **Table S2.** PAT-Seq mapped data. **Table S6.** Analysis of enriched motifs in pharynx and body muscle promoters. Additional file 2: Table S3. Genes expressed in each tissue dataset. Additional file 3: Table S4. Transcription factors expressed exclusively in each tissue profiled in this study. Additional file 4: Table S5. mRNA splice isoforms that significantly changing in expression level between tissues. Additional file 5: Table S7. 3’UTR isoform changes between tissues. Additional file 6: Table S8. Primers used in this study.

## Abbreviations

3’UTR: 3’ untranslated region
APA: alternative polyadenylation
bp: base pair
CPSF: cleavage and polyadenylation specificity factor
CstF: cleavage stimulation factor
FPKM: fragments per kilobase per million
GFP: green fluorescent protein
kb: kilobase
miRNA: microRNA
NGM: nematode growth medium
PABPC: cytoplasmic poly(A) binding protein
PAS: polyadenylation signal
PAT-Seq: polyA-tagging and sequencing
PBS: phosphate-buffered saline
PCR: polymerase chain reaction
snRNP: small nuclear ribonucleoprotein
