# Supplementary material for: Comparative RNA-Seq analysis reveals pervasive tissue-specific alternative polyadenylation in Caenorhabditis elegans intestine and muscles
Source: BMC Biol. 2015 Jan 20;13:4. doi: 10.1186/s12915-015-0116-6 (PMC4343181; doi:10.1186/s12915-015-0116-6)
Supplement: Additional file 1: Figure S1. — PAT-Seq sequencing results. Figure S2. Validation of tissue-specific genes detected by PAT-Seq. Figure S3. Comparative analysis with other available intestine-enriched datasets. Figure S4. Differential mRNA isoform expression analysis. Figure S5. Sequence analysis of promoter regions for intestine expressed genes. Figure S6. Analysis of PAS usage between tissues. Figure S7. PAS location and sequence requirement. Table S1. PAT-Seq raw sequencing data. Table S2. PAT-Seq mapped data. Table S6. Analysis of enriched motifs in pharynx and body muscle promoters. [file 12915_2015_116_MOESM1_ESM.pdf]

Additional File 1 for:

Comparative RNA-Seq analysis reveals pervasive tissue-specific alternative polyadenylation in *C. elegans* intestine and muscles

Stephen M Blazie, Cody Babb, Henry Wilky, Alan Rawls, Jin G Park and Marco Mangone*

* To whom correspondence should be addressed. Tel: (480) 965-7957; Email: mangone@asu.edu

This PDF includes:

Figures S1-S7

Table S1 – PAT-Seq raw sequencing data.

Table S2 – PAT-Seq mapped data.

Table S6 – Analysis of enriched motifs in pharynx and body muscle promoters.

Table of Contents
Additional File 1

Figure S1: PAT-Seq sequencing results. 3

Figure S2: Validation of tissue-specific genes detected by PAT-Seq. 4

Figure S3: Comparative analysis with other available intestine-enriched datasets. 5

Figure S4: Differential mRNA isoform expression analysis. 6

Figure S5: Sequence analysis of promoter regions for intestine expressed genes. 7

Figure S6: Analysis of PAS usage between tissues. 8

Figure S7: PAS location and sequence requirement. 9

Table S1: PAT-Seq raw sequencing data. 10

Table S2: PAT-Seq mapped data. 10

Table S6: Analysis of enriched motifs in pharynx and body muscle promoters. 11

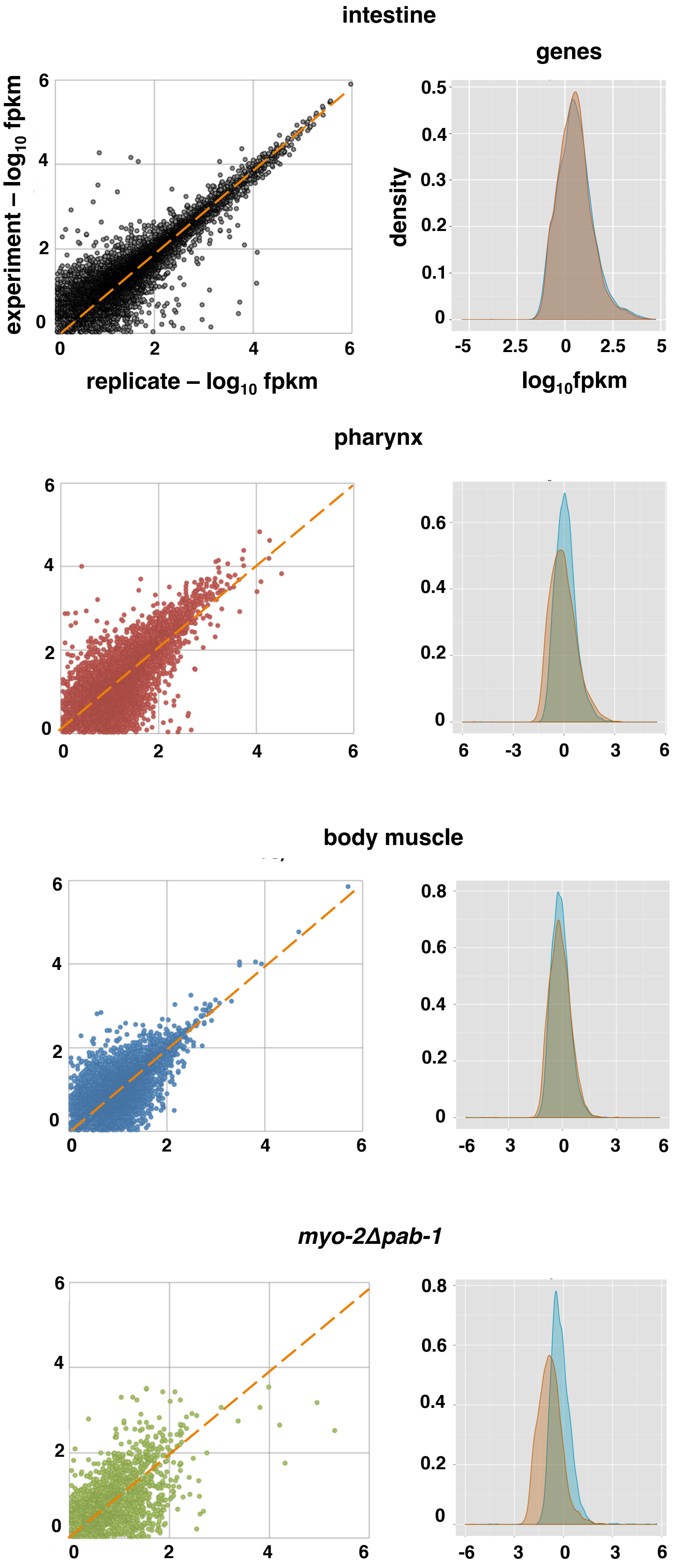

Figure S1: PAT-Seq sequencing results. Scatter plot of mapped genes from each tissue dataset displayed by fpkm value detected in each replicate on a logarithmic (log_{10}) scale to highlight similarity of detection between replicates. The trendline (yellow) displays the expected distribution for 100% similarity between replicates. The right panels show the distribution of the fpkm values in control and replicate samples for each tissue. The plots were generated using the cummeRbund package v. 2.0.

Supplementary Figure 2

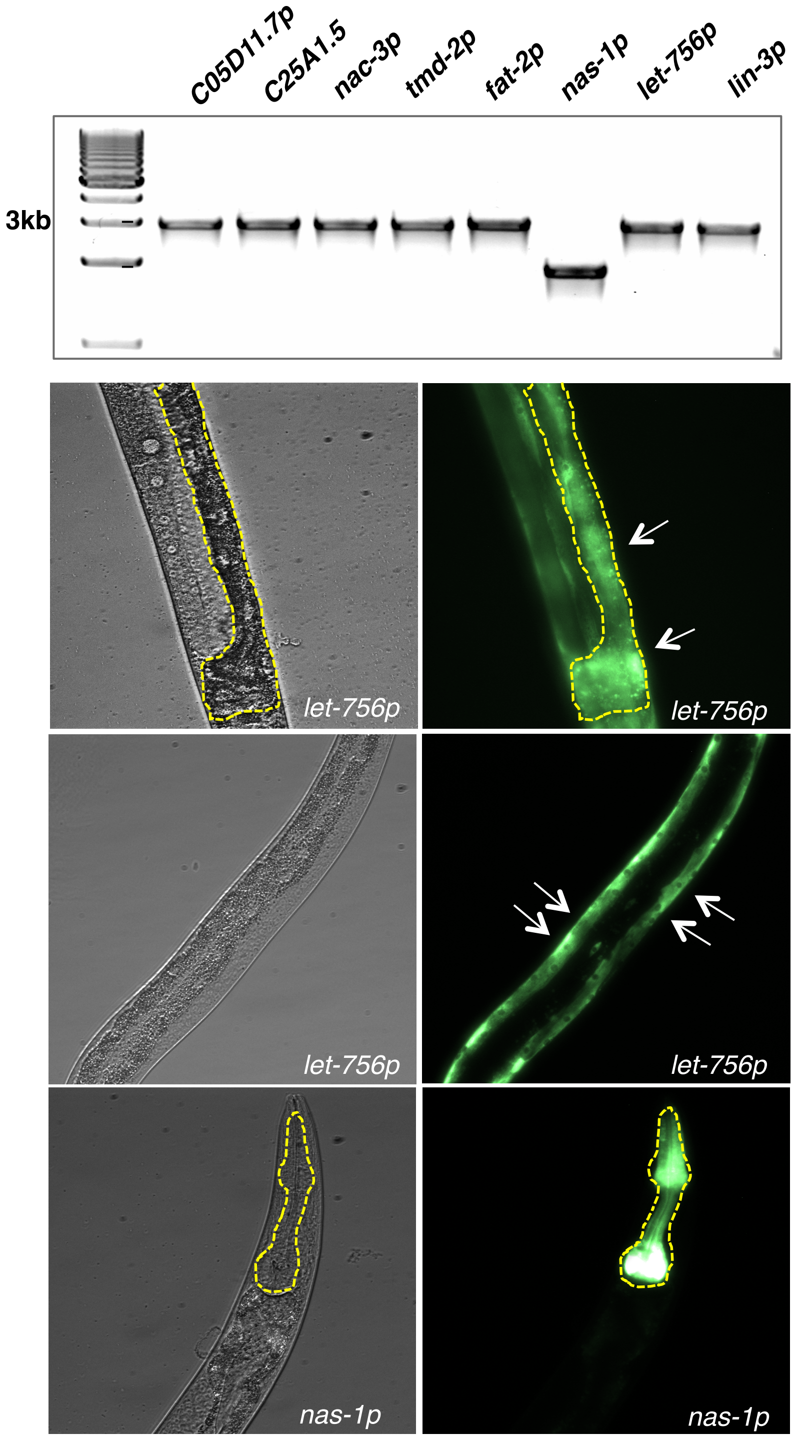

gene	intestine		pharynx		Body muscle	
	detected	validated	detected	validated	detected	validated
C05D11.7	*	yes	*	yes	*	yes
C25A1.5	**	yes	*	yes	*	yes
nac-3	*	yes	*	yes	*	yes
tmd-2	*	no	*	yes	*	yes
fat-2	***	yes	**	yes	*	yes
nas-1	-	-	*	yes	-	-
let-756	*	yes	*	yes	*	yes
lin-3	-	-	*	yes	*	no

putative expression index:
*- not detected; * low expression; ** expressed; *** strong expression*

Figure S2: Validation of tissue-specific genes detected by PAT-Seq. We have cloned promoter regions for eight tissue-specific genes and used them to drive *in vivo* expression of our PolyA-Pull plasmid containing GFP. Top Panel: Electrophoresis results of PCR confirming the cloning of each of eight promoters upstream of GFP. Middle Panel: Three selected images of transgenic worms expressing GFP in intestine (top panel) and body muscle (middle) driven by *let-756* promoter, and pharynx (bottom) driven by *nas-1* promoter. Bottom Panel: Table displaying comprehensive results for each of eight promoters driving expression of GFP *in vivo*. The putative expression index reflects the level of expression of each gene obtained from PAT-Seq data in each tissue.
We validated gene expression in 19/21 cases using this strategy. Seven out of eight of these genes were detected by our approach in all three tissues. Importantly, the strength of the GFP signal detected in most of the tissues correlate with the expression levels from our sequencing data (data not shown).

Out of 21 total experiments, all but two cases were confirmed by GFP expression in the correct tissue (19/21, ~90% of cases). We were unable to detect expression data in the correct tissue for *tmd-2* (intestine) and *lin-3* (body muscle). However these genes may be expressed below the limit of GFP detection. Together, these results provide evidence that PAT-Seq is indeed a sensitive and specific technique to enrich for tissue-specific mRNAs in worms.

A

datasets	# of genes
WS190	28,122
this study (intestine)	7,361
Haenni *et al.*	3,502
Pauli *et al.*	1,647
McGhee *et al.*	5,623

B

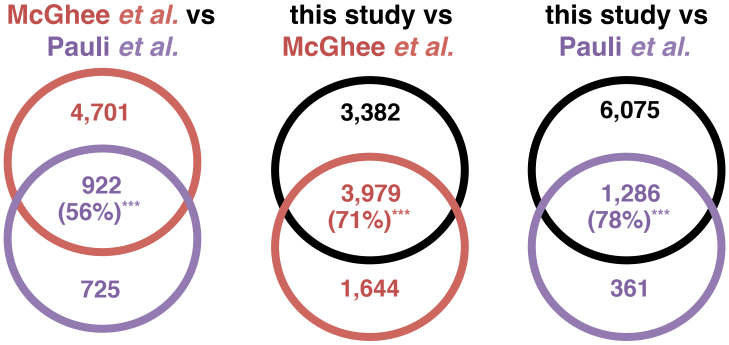

C

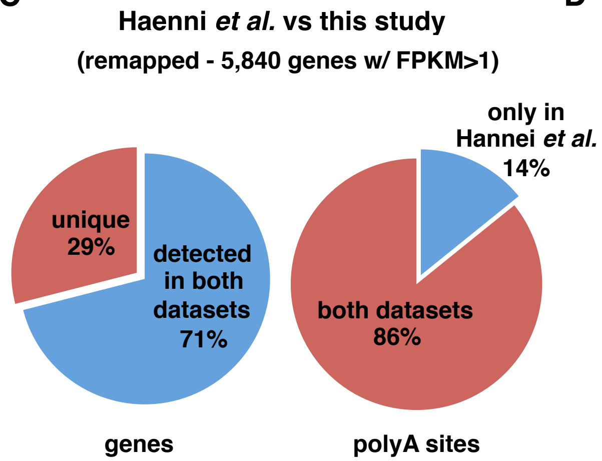

D

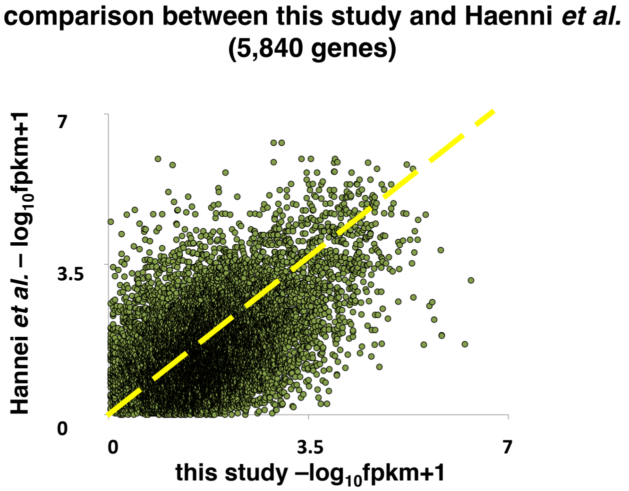

E

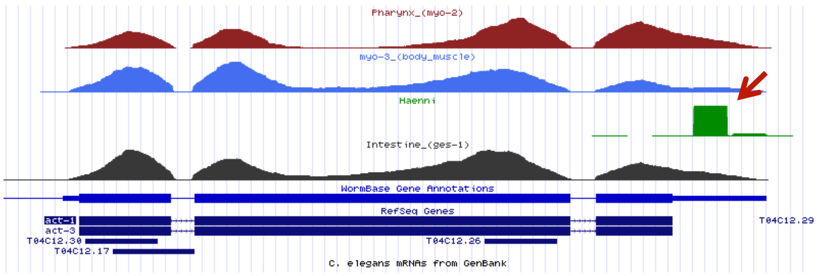

F

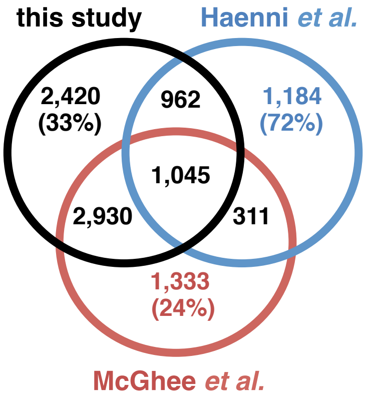

Figure S3: Comparative analysis with other available intestine-enriched datasets. We have compared our intestine dataset with Haennei *et al.*, Pauli *et al.,* and McGhee *et al.* (*A*) Summary tables with total number of genes detected in each dataset. (*B*) The overlaps between our intestine dataset (this study) and McGhee and Pauli datasets. Only 56% of genes present in McGhee *et al.* and Pauli *et al.* overlap. Our intestine dataset instead overlaps with these two datasets 71% and 78% respectively. ***P<0.001, by one-tailed randomization test (N=1,000). (*C*) We downloaded and remapped the raw data from the 'sorted' dataset produced by Haenni et al., and studied its degree of correlation with our dataset (this study). Left: 71% of the top 1,000 genes from the remapped Hannei et al. dataset overlap with intestine genes detected from our study, whereas 29% of genes are only detected in the remapped Haenni et al., dataset. Right: 86% (n=6,316) of the polyA sites detected by this study overlap with 3'UTR ends remapped from the Hannei et al. intestine dataset. (*D*) Comparison of gene expression levels between genes expressed in both Haenni *et al.*, and our intestine dataset. (*E*) Example of gene expression coverage in our dataset (this study) vs Haenni *et al.*, who used an approach that only mapped the 3'ends of transcripts (red arrow) that are bioinformatically attached to the closest gene model. (*F*) The overlap between genes detected in our intestine dataset (this study), Haenni *et al.* (not remapped), and McGhee *et al.* datasets. We detected a core set of 1,045 genes that are identified by all three datasets.

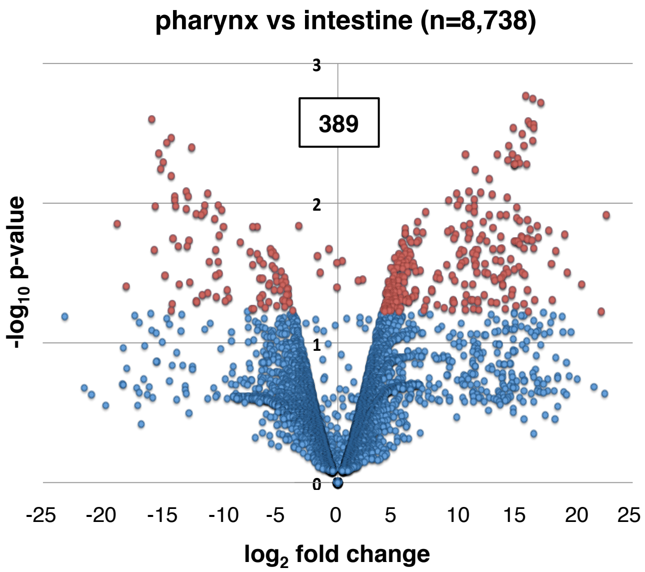

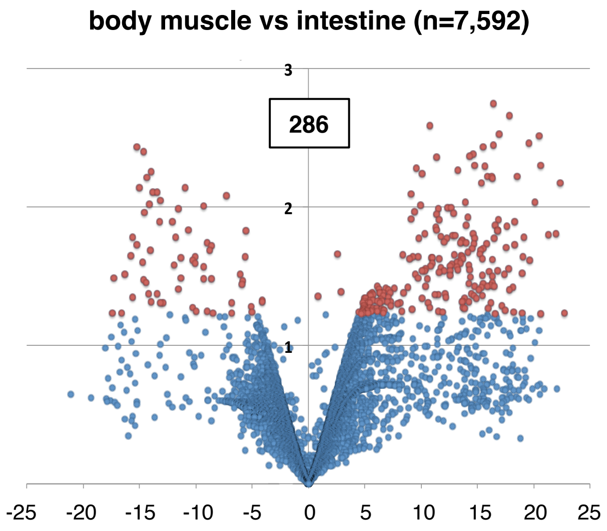

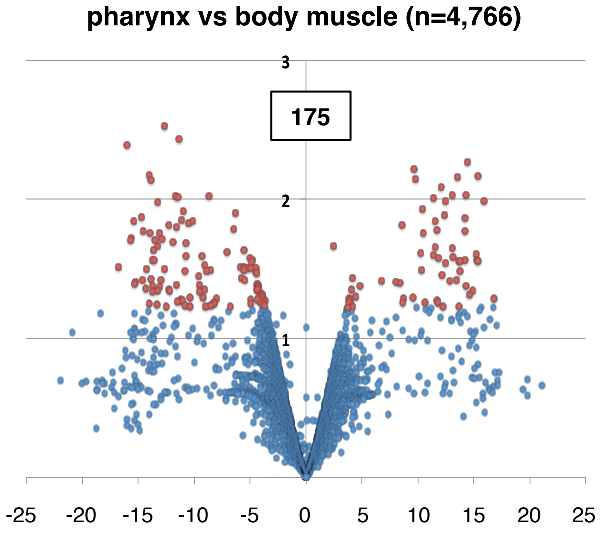

Figure S4: Differential mRNA isoform expression analysis. We have studied the changes in mRNA splice isoform expression for genes detected among each combination of two tissues in our datasets. Volcano plots showing the changes of isoform expression between each tissue (p-value versus fold-change). Total number of isoforms that significantly switch between two tissues ($p<0.05$) are shown in red and the total number of genes in this category are boxed. A list of all isoforms identified are included in Supplementary Table 5.

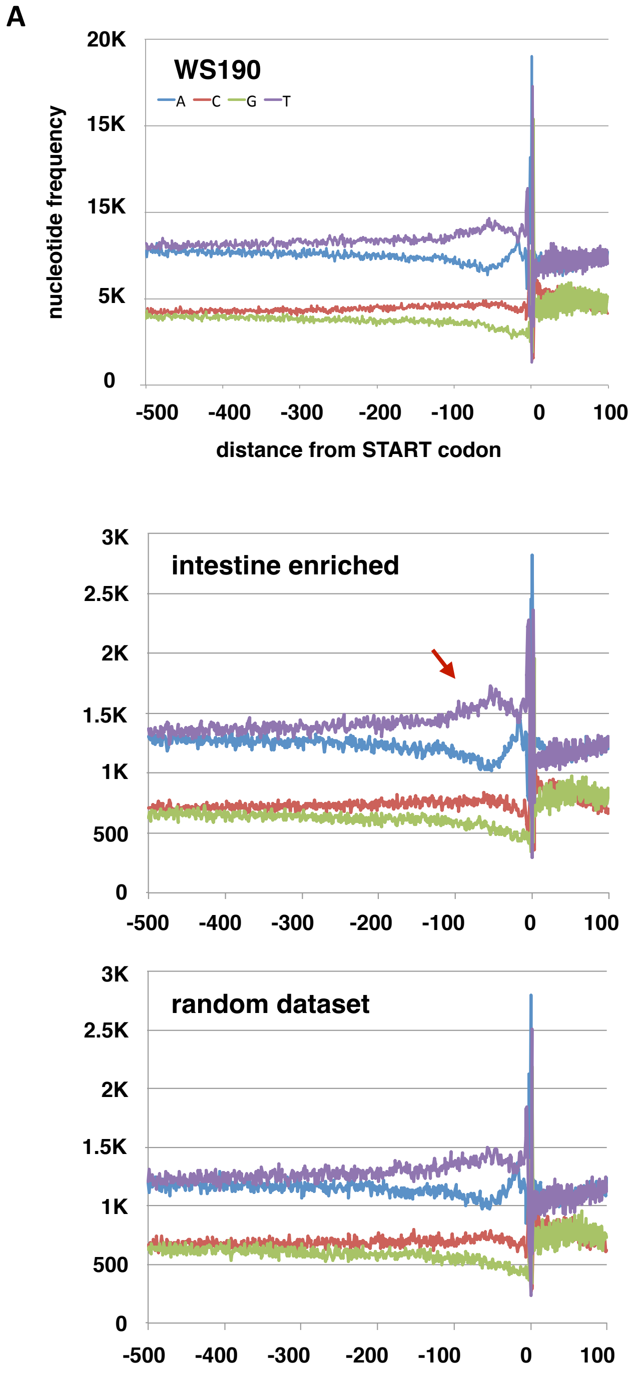

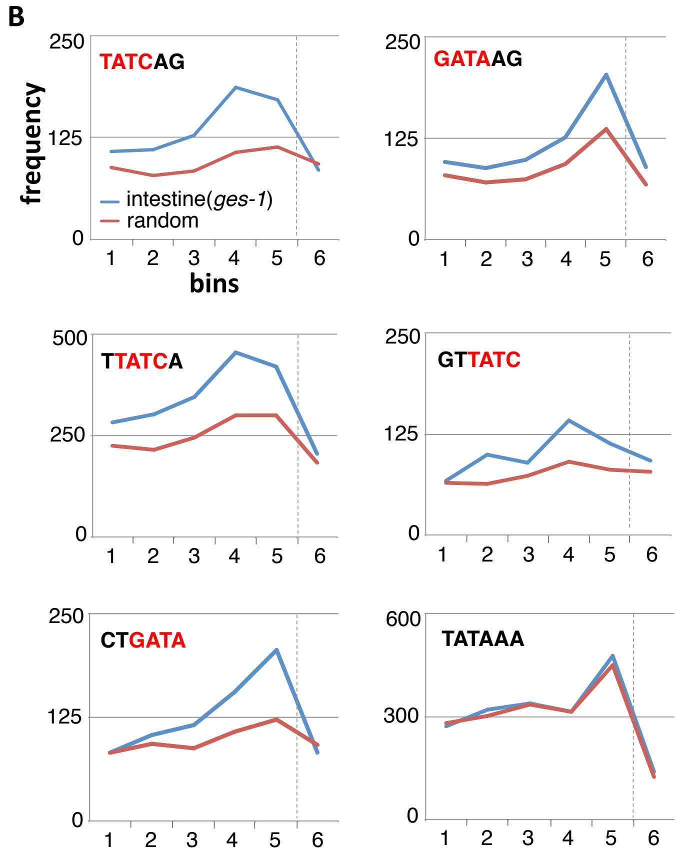

C

motif	random dataset	intestine enriched (% increase)
GATACG	200	271 (+36%)***
CGATAA	523	699 (+34%)***
GATAAG	560	751 (+34%)***
CGATAA	523	699 (+34%)***
CTTATC	698	923 (+32%)***
GCTATC	212	275(+30%)***
ACGATA	368	478 (+30%)***

Figure S5: Sequence analysis of promoter regions for intestine expressed genes. We extracted and studied the DNA regions 500bp upstream from the start codon for each of 4,095 genes unique in our intestine dataset. (*A*) The average base composition for promoter regions in all WS190 transcripts (top), intestine-specific transcripts (middle), and a random dataset of 4,095 genes (bottom). We detected a strong enrichment of tymidine within 100nts upstream of the transcription start site (red arrow). (*B*) Enrichment of hexamers with the conserved 'GATA' or its antisense 'CTAT' element in promoters from genes uniquely expressed in intestine compared with the same number of randomly selected promoters. The canonical 'TATAAA' (TATA-box) was used as a comparison (bottom right) to show equal enrichment of this hexamer in both intestine and random sets. (*C*) Hexamers with the conserved 'GATA' element and the percent enrichment of this element in our intestine dataset over the set of randomly selected genes. ***P<0.001, by 2-tailed Chi-square test.

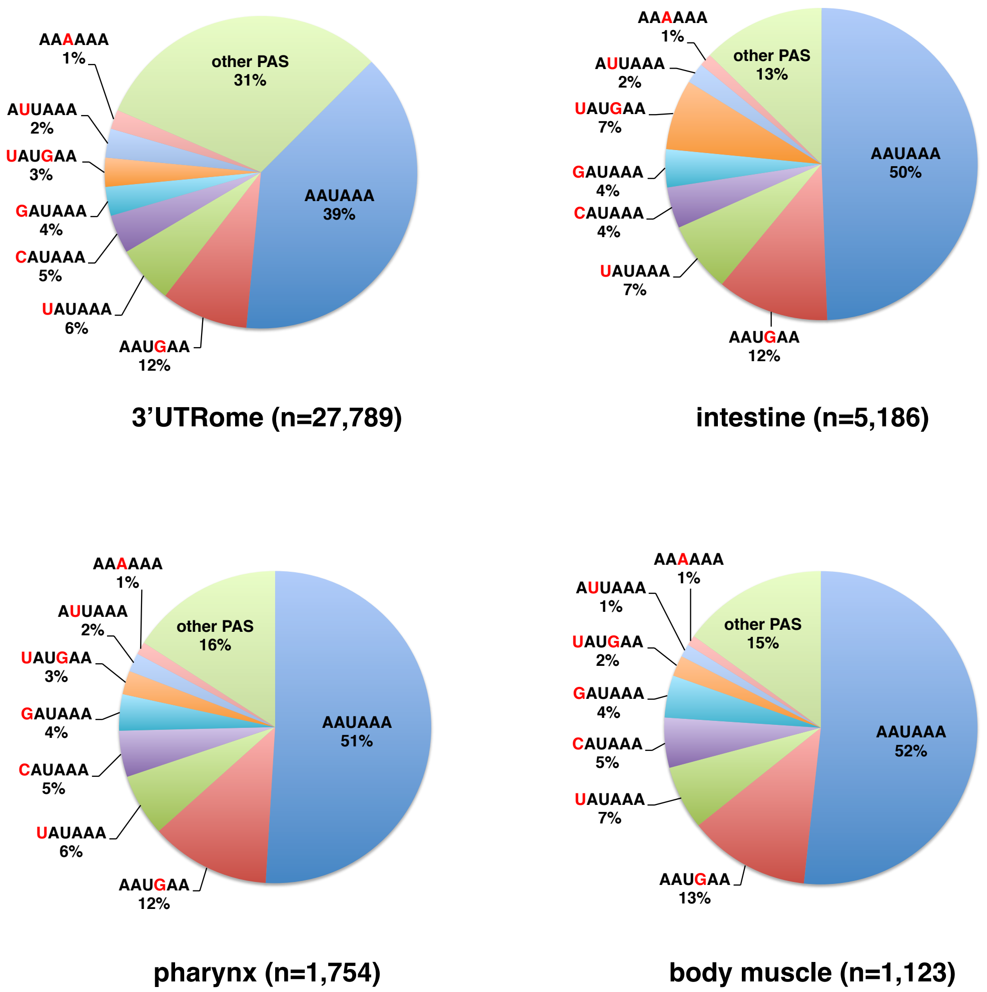

Figure S6: Analysis of PAS usage between tissues. We extracted the PAS elements present in the 3'UTRome and assigned them to 3'UTR isoforms present in each of our tissue datasets. Each chart represents the percentage of distinct isoforms present in each tissue dataset containing the canonical PAS 'AAUAAA' (blue), seven of the next most common PAS elements, and the remaining 'other PAS' sites. The nucleotide changes from the canonical PAS element are highlighted in red.

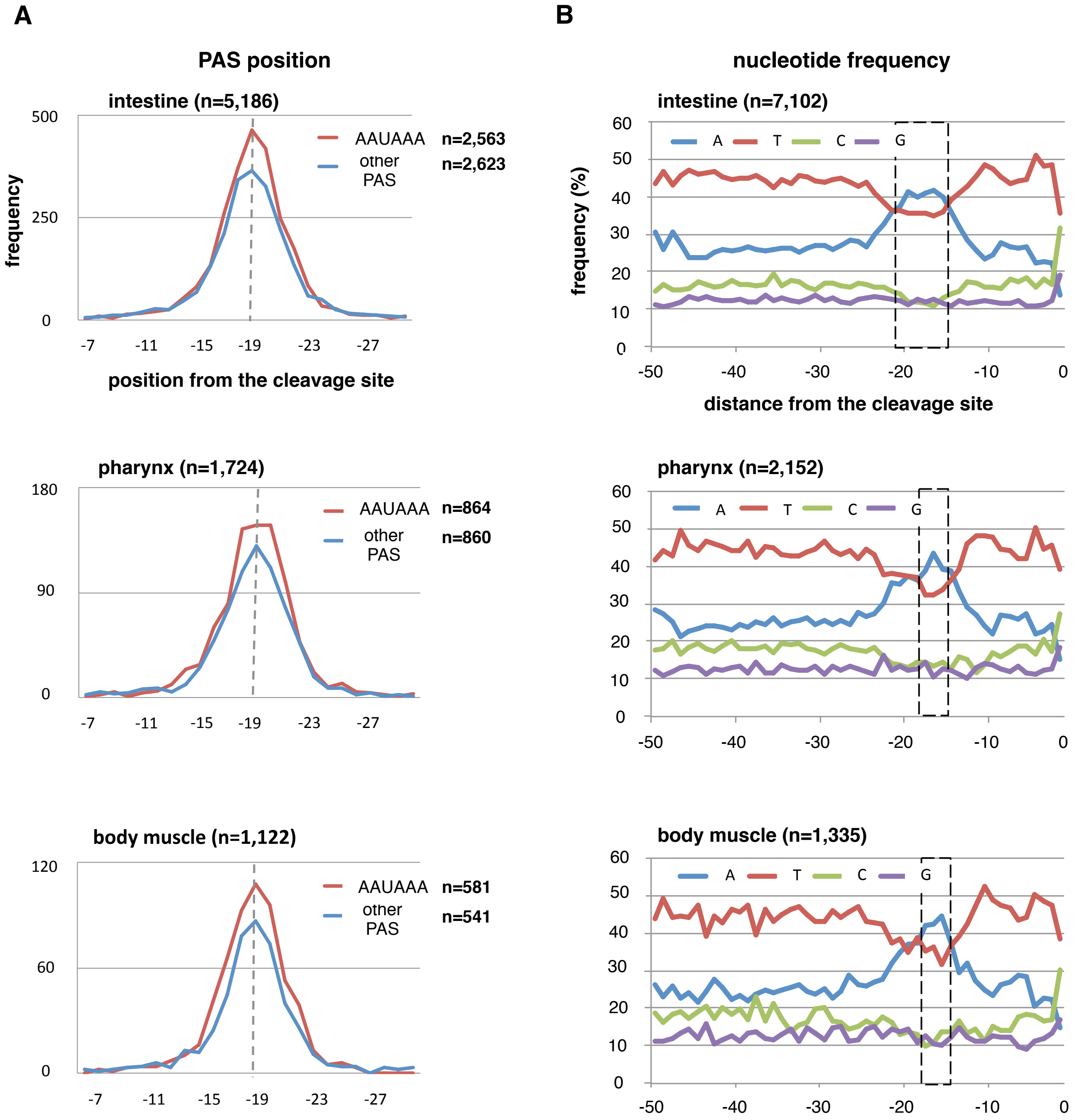

Figure S7: PAS location and sequence requirement. We assigned a PAS element to each 3'UTR isoform using data from the 3'UTRome database (Mangone *et al.*) and studied the tissue-specific changes in PAS hexamers in genes in intestine, pharynx, and body muscle. (*A*) Plot showing the position of the canonical PAS 'AAUAAA' and other PAS sites with reference to the cleavage site for all isoforms in each tissue for 3'UTRs in common with Mangone *et al.* All three tissues have a strict positional requirement for PAS elements at -19nts from the cleavage site. (*B*) Nucleotide frequency distribution within the cleavage sites. While all these tissues have a very similar pattern, intestine genes have an unusual larger adenosine-enriched block at -20nts from the cleavage site (dashed box).

samples (tissue)		total reads	mapped (%)	not mapped	average depth
intestine	*experiment*	18,148,228	11,473,900 (63%)	6,674,328	65.2x
	replicate	14,948,680	9,376,501 (63%)	5,572,179	49.6x
pharynx	*experiment*	15,685,384	12,028,022 (77%)	3,657,362	17.4x
	replicate	14,798,098	10,370,652 (70%)	4,427,446	28.5x
body muscle	*experiment*	15,496,850	10,818,093 (70%)	4,678,757	9.8x
	replicate	16,885,324	12,829,775 (80%)	4,055,549	12.1x
myo-2Δpab-1 (- control)	*experiment*	13,644,473	10,589,072 (78%)	3,055,401	51.2x
	replicate	18,703,551	15,863,763 (85%)	2,839,788	18.2x

Table S1: PAT-Seq raw sequencing data. Raw reads derived from tissue-specific mRNA libraries on the Illumina Hi-Seq Instrument, mapped to the *C. elegans* WS190 genome annotation.

samples (tissue)		genes		isoforms	
intestine	*experiment*	7,971	7,355(*)	8,987	8,519(*)
	replicate	8,254		9,432	
pharynx	*experiment*	4,188	3,094(*)	4,427	3,650(*)
	replicate	3,998		4,362	
body muscle	*experiment*	3,404	2,604(*)	3,610	3,024(*)
	replicate	3,478		3,679	
myo-2Δpab-1 (- control)	*experiment*	796	1,011(*)	826	1,120(*)
	replicate	1,146		1,247	

Table S2: PAT-Seq mapped data. Mapped reads from the tissue-specific mRNA libraries on the Illumina Hi-Seq Instrument. Genes and isoforms are mapped to the *C. elegans* WS190 genome annotation. Genes and isoforms marked with an asterisk correspond to genes and isoforms enriched in both biological duplicates.

intestine

#	Motif	Logo	RC Logo	*E*-value	TF (*p*-value)
1	CTGAAAWW	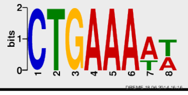	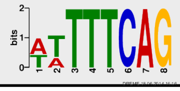	8.9e-183	–
2	ATATWTW	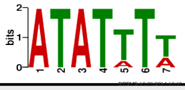	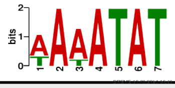	4.7e-094	–
3	CHCCDCC	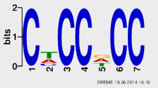	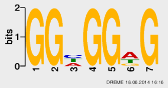	2.4e-093	KLF5 (0.0003)<br>SP1 (0.0005)<br>EGR1 (0.0001)<br>ZNF263 (0.0001)<br>ZNF354C (0.002)<br>SP2 (0.003)
4	STACNGTA	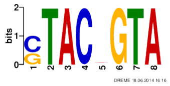	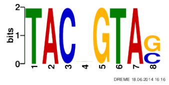	4.8e-081	–
5	GAGARRA	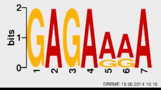	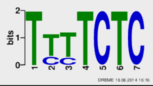	2.5e-077	–
6	CANTTTYC	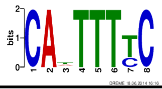	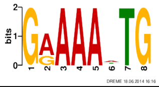	2.1e-059	–
7	GAGACSCA	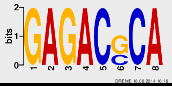	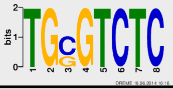	3.0e-047	EOR-1* (0.0001)<br>GATA1 (0.0005)
8	ACTGATAR	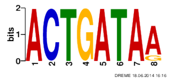	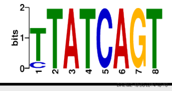	5.1e-044	–
9	GAGCWS	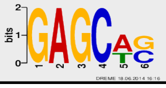	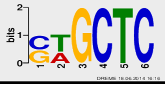	1.7e-043	–
10	ADAAATA	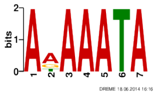	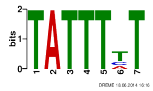	3.1e-043	MEF2C<br>(4.75125e-06)<br>MEF2A<br>(4.75125e-06)
11	DCTGRAA	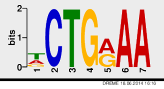	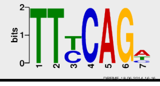	1.3e-036	–
12	AAAATGT	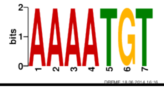	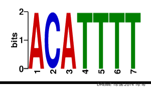	2.4e-033	–

Table S6: Analysis of enriched motifs in pharynx and body muscle promoters. We have used the MEME Suite software to compare tissue-specific enrichment of our top motifs (p-value <.005). Human transcription factor orthologs are shown in the last column. * worm genes

pharynx

#	Motif	Logo	RC Logo	*E*-value	TF (*p*-value)
1	AATATWK	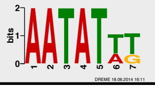	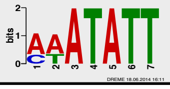	8.5e-010	–
2	CTCCNCC	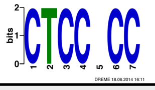	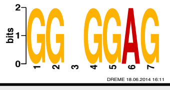	5.5e-009	ZNF263 (0.0003)
3	ASAAGAAG	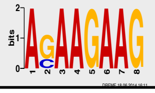	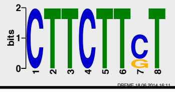	4.2e-007	–
4	AWTTTCAG	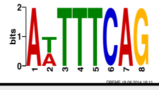	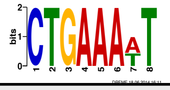	4.2e-007	–
5	ASTGTR	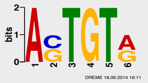	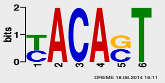	1.1e-006	–
6	AAAAYTR	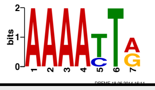	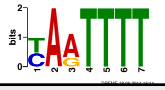	7.4e-004	–
7	AGAGASR	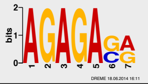	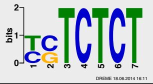	7.4e-004	EOR-1* (0.0001)
8	CAACKACA	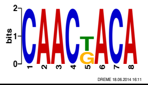	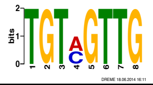	1.2e-003	–
9	CCGCCB	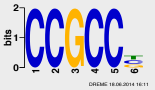	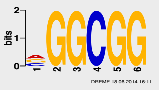	2.5e-003	SP1 (0.0006)<br>SP2 (0.0001)<br>E2F3(0,001)<br>E2F6 (0.0008)<br>EGR-1 (0.0009)
10	AAAATGVA	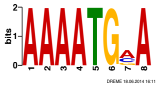	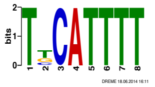	7.5e-003	STAT1 (0.0001)<br>IRF1 (0.0009)<br>BLMP-1* (0.001)<br>SKN-1* (0.001)<br>PRDM1 (0.001)
11	CTRGAA	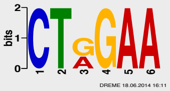	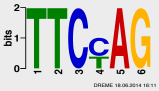	2.3e-002	HSF1 (0.0005)<br>STAT1 (0.0008)
12	GGAGSCA	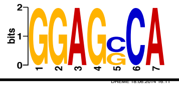	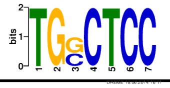	4.7e-002	–

body muscle

#	Motif	Logo	RC Logo	*E*-value	TF (*p*-value)
1	CTCCDCC	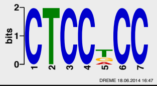	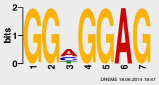	2.8e-008	–
2	CAKTTTTC	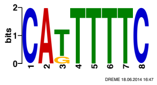	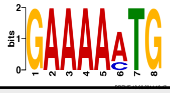	2.2e-007	–
3	AWATATW	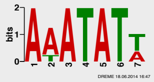	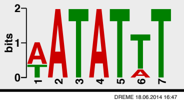	2.8e-007	–
4	CGYCGYC	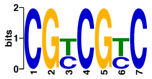	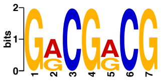	3.5e-006	–
5	TCTGRAA	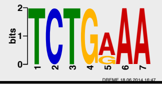	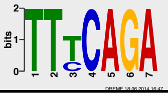	1.9e-005	–
6	AGAAGRAG	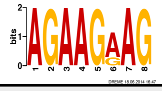	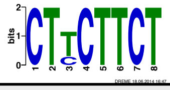	7.5e-005	–
7	TACBGTA	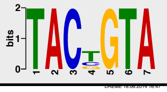	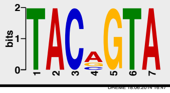	2.0e-003	–
8	GGCGGS	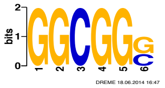	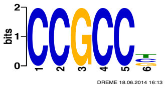	2.3e-003	E2F1 (0.0001)<br>E2F4 (0.0001)<br>E2F6 (0.0002)<br>SP2 (0.0004)<br>EGR1 (0.0004)<br>SP1 (0.0005)
9	AAAAASTG	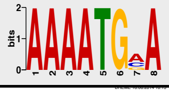	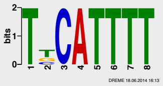	4.5e-003	–
10	GAGACRCA	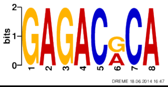	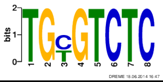	5.2e-003	EOR-1*<br>(2.5097e-05)
11	CACCAC	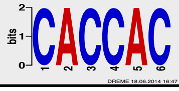	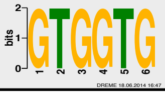	2.0e-002	–
12	GMGCACA	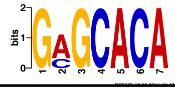	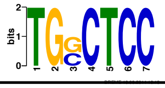	4.8e-002	–

intestine vs pharynx

#	Motif	Logo	RC Logo	*E*-value	TF (*p*-value)
1	KCTTATCA	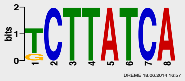	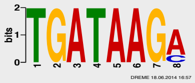	1.9e-003	ELT-3* (2.52093e-06) GATA3 (0.0003) GATA2 (0.0005)
2	AATAAY	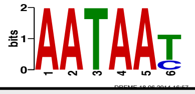	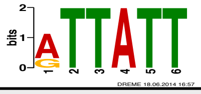	1.3e-002	–
3	AWTCAATA	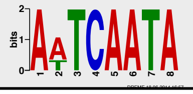	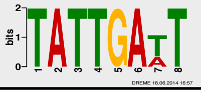	2.3e-002	–
4	DTATTTCA	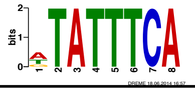	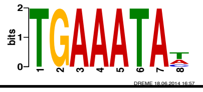	2.9e-002	–

intestine vs body muscle

#	Motif	Logo	RC Logo	*E*-value	TF (*p*-value)
1	CTGATAR	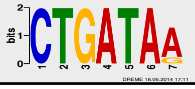	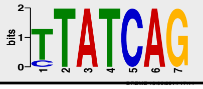	3.4e-006	GAT1 (0.0002)
2	ASAAATA	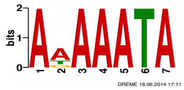	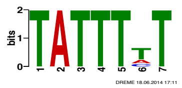	6.4e-003	MEF2A (1.10537e-06) MEF2C (6.4933e-06)

pharynx vs intestine

#	Motif	Logo	RC Logo	*E*-value	TF (*p*-value)
1	CTTCTTCK	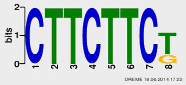	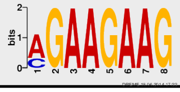	3.9e-003	–
2	TCATCCB	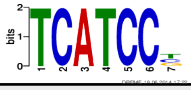	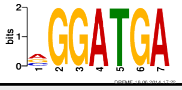	4.9e-003	–
3	GATGGAGC	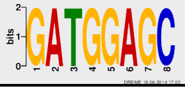	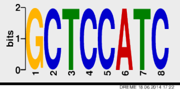	2.1e-002	–
4	ACTTACST	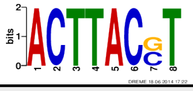	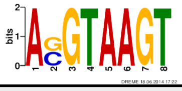	2.5e-002	–
5	CCCCK	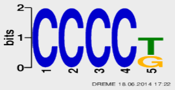	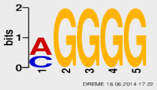	4.6e-002	PLAG1 (6.9393e-05) MZF1 (0.001)

body muscle vs intestine

#	Motif	Logo	RC Logo	*E*-value	TF (*p*-value)
1	GGARGW	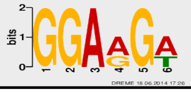	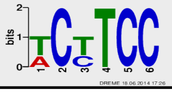	5.2e-003	–
2	AGCATAKC	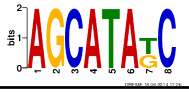	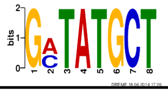	1.7e-002	–
